# An integrative probabilistic model for identification of structural variation in sequencing data

**DOI:** 10.1186/gb-2012-13-3-r22

**Published:** 2012-03-27

**Authors:** Suzanne S Sindi, Selim Önal, Luke C Peng, Hsin-Ta Wu, Benjamin J Raphael

**Affiliations:** 1 Center for Computational Molecular Biology, Brown University, Box 1910, Providence, RI 02912, USA; 2 Department for Molecular Biology, Cellular Biology and Biochemistry, Brown University, 185 Meeting St, Providence, RI 02912, USA; 3 Department of Computer Science, Brown University, 115 Waterman St. Providence, RI 20912, USA

## Abstract

Paired-end sequencing is a common approach for identifying structural variation (SV) in genomes. Discrepancies between the observed and expected alignments indicate potential SVs. Most SV detection algorithms use only one of the possible signals and ignore reads with multiple alignments. This results in reduced sensitivity to detect SVs, especially in repetitive regions. We introduce GASVPro, an algorithm combining both paired read and read depth signals into a probabilistic model that can analyze multiple alignments of reads. GASVPro outperforms existing methods with a 50 to 90% improvement in specificity on deletions and a 50% improvement on inversions. GASVPro is available at http://compbio.cs.brown.edu/software.

## Background

Structural variation, including duplications, deletions and rearrangements of large blocks of DNA sequence, is now recognized as an important contributor to the genetic differences between individual humans and the somatic differences between normal and cancer cells [1-7]. It is also prevalent in other organisms, including many model organisms [8-10]. Knowledge about the extent of structural variation has increased rapidly in the past few years with improvements in DNA microarray and sequencing technologies. In particular, sequencing approaches identify all types of structural variation, including copy number variants and balanced rearrangements like inversions and reciprocal translocations [11-13]. While next generation sequencing technologies are now widely used to assess both genetic variation in normal genomes [14-21] and somatic structural variation in cancer genomes [4,7,22,23], the short reads and short inserts of these technologies make the identification of many structural variants (SVs) non-trivial. Since *de novo *assembly of mammalian genomes from next-generation sequencing technologies remains a challenge [24,25], many SVs are identified using a resequencing approach where sequence reads from an individual genome are aligned to a reference human genome assembly. The resequencing approach thus leverages the extensive finishing efforts employed in the generation of the human reference genome.

Many strategies have been employed to predict structural variation using the resequencing approach [11-13]. First, read depth (RD), the density of mapped reads to an interval of the reference genome, has been used successfully to identify copy number variants [26-31]. However, RD is unable to detect copy neutral variants such as inversions and balanced translocations. Second, paired read (PR) approaches have been used to identify all types of SVs, both copy number variants and copy-neutral variants [16,28,32-35]. These approaches analyze the collection of PR mappings and find clusters of aberrantly mapped PRs that suggest SVs distinguishing the two genomes. Third, split read (SR) methods have been employed to directly identify sequence reads that contain breakpoints of SVs [36]. However, the short reads produced by current second-generation sequencing technologies have limited the use of SRs for SV detection; for example, Ye *et al*. [36] rely on anchoring the search for SRs using a full-length alignment of one read from a PR.

While there has been extensive development of methods for structural variation prediction, there remains room for improvement. First, most existing methods for SV prediction use only one of the possible signals (RD, PR or SR). A few methods employ a second signal in later post-processing of predictions. Such a *post hoc *approach may improve specificity, but it does not increase sensitivity by combining multiple, weak signals. Although a few recent methods have begun to consider both RD and PR signals [37,38], these methods have focused only on copy number variants. Second, most methods for structural variation prediction used only reads with unique high-confidence alignments to the reference genome, ignoring reads with lower quality alignments or multiple possible alignments [32,33,39]. As such, these methods have very low sensitivity to detect repeat-associated rearrangements. Since many SVs are associated with repetitive sequences, including segmental duplications [40], and mobile elements [2], a substantial improvement in sensitivity may be possible by including reads with multiple alignments. More recently, a few methods have been introduced that consider multiple or lower quality alignments of reads relying on various criteria to select among possible candidate alignments [34,41,42]. While these methods may predict more true variants, this increased sensitivity often comes at the cost of reduced specificity as these methods produce many false positive predictions. Thus, there is a need for additional improvements in sensitivity and specificity for SV prediction. For example, the pilot study of the 1000 Genomes Project did not report inversion SVs [43] even though such variants have been previously shown to be abundant in normal genomes [16].

Here, we introduce GASVPro, an algorithm for SV identification that integrates both RD and PR signals into a unified probabilistic model. We find that the likelihood of a predicted variant under our probabilistic model provides a better criteria for prioritizing predictions than the number of supporting PRs, a common heuristic for ranking predictions. In addition to combining both RD and PR signals, GASVPro explicitly reports uncertainty in each predicted breakpoint, which is useful information for identification of SRs or designing assays for experimental validation. This breakpoint localization is obtained using a computational geometric algorithm, Geometric Analysis of Structural Variants (GASV) [33], that represents all possible breakpoints, or breakends, that are consistent with the aligned reads as a polygon in two-dimensional genome space. By carefully clustering only those PRs that genuinely support the same breakends, GASV avoids over-collapsing fragments into the same SV prediction, a problem demonstrated in other methods (see Results) and reports coordinates consistent with the true variant points.

Moreover, GASVPro exploits this explicit representation of the breakends to incorporate a subtle signal of highly localized drops in coverage at the variant breakends. We call this signal breakend read depth (beRD), and it occurs for both copy number variants as well as copy-neutral SVs. Using this signal, GASVPro predicts whether a generic breakend is a homozygous or a heterozygous variant, even when relatively few PRs support the variant. Thus, GASVPro is the first method to utilize RD to predict generic SVs, including inversions and reciprocal translocations, and not just copy number variants. For deletions, GASVPro uses the stronger signal of RD across the entire deleted interval, and this combination of PR and RD leads to highly sensitive and specific deletion predictions. GASVPro also considers reads with multiple possible alignments, using a Markov chain Monte Carlo (MCMC) approach to sample over the space of possible mappings for each paired-end sequenced fragment. In this way, GASVPro does not select only a single 'best' alignment for each fragment, but rather computes a posterior probability of each variant over all possible alignments of each read.

We demonstrate the advantages of GASVPro on simulated data and Illumina sequencing data from two sequenced human genomes, NA18507 [14] and NA12878 [44] (1000 Genomes Project). We compare predictions to known variants with a novel metric, the 'double uncertainty' metric, developed to allow for unambiguous comparisons when there is uncertainty in the breakpoint locations. For deletions, GASVPro outperformed competing methods by attaining equal or greater sensitivity while making at least 50% and up to 90% fewer predictions. In addition, on a subset of deletions with known ploidy, GASVPro successfully classifies over 85% as homozygous or heterozygous. For inversions, GASVPro is up to twice as specific at maximum sensitivity than existing methods. In particular, because of GASVPro's use of the beRD signal, it is the only method to attain optimal specificity and sensitivity on our simulated data set. In other cases, GASVPro's use of the beRD signal at inversion breakpoints results in equal or better specificity than competing methods despite considering a larger set of possible alignments.

## Results

### A probabilistic model of structural variant breakends

#### Identifying structural variants from paired-read sequencing data

**Figure 1:**
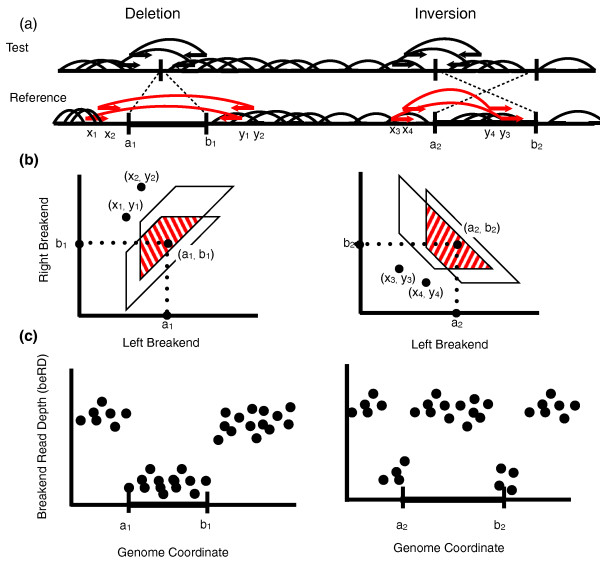
**Signals of structural variation from paired-end sequencing**. **(a) **Fragments (black arches) from a test genome are sequenced from both ends and the resulting paired reads are mapped to a reference genome. Fragments containing the breakpoint of a structural variant (black arches with arrows) have a discordant mapping (red). **(b) **The GASV program [33] efficiently clusters discordant fragments supporting the same variant and provides precise information about the localization of the adjacency, (*a*,*b*), created by the rearrangement. For example, on the left a deletion of the interval $\left[a_{1}+1,b_{1}-1\right]$ from the reference creates a novel adjacency $\left(a_{1},b_{1}\right)$ of breakends $a_{1}$ and $b_{1}$. GASV represents the novel adjacency as a breakend polygon (shaded red trapezoid) where the left and right breakends of the variant must lie within the breakend polygon. In this example, we show breakend polygons for a deletion (left) and an inversion (right), each supported by two discordant fragments. **(c) **The presence of a structural variant is also indicated by changes in the depth of coverage of concordant mappings. For the deletion (left) the depth of coverage is low throughout the entire region, while for the inversion (right) the depth of coverage drops only near the breakends.

In PR mapping, fragments from a test genome are sequenced from both ends and the resulting PRs are aligned to a reference genome. The goal of the alignment process is to determine the correct mapping of the fragment, that is, the corresponding position of the fragment in the reference genome (Figure 1a). For now, we assume that all reads have a single high-quality alignment to the reference, which corresponds to its mapping, and consider the problem of reads with multiple alignments later.

Although the length of each individual fragment is generally unknown, the size selection that is performed during the construction of the sequencing library yields an approximate distribution of fragment lengths. We assume that fragment lengths are between $L_{\text{min}}$and $L_{\text{max}}$; these values can be derived from the empirical distribution of mapped fragments. Fragments with both ends mapping uniquely to the reference with 'convergent orientation' and with mapped distance in the range $\left[L_{\text{min}},L_{\text{max}}\right]$ are called concordant fragments because their mapping indicates concordance (no SV) between the test and reference genome. (We note that the definition of convergent orientation depends on sequencing technology. For example, with Illumina paired-end data, the reads are obtained from opposite DNA strands and thus convergent orientation is defined as reads with opposite orientation, with the left read forward and the right reversed (+/-). With SOLiD paired-end data, reads are obtained from the same DNA strand and thus should have the same orientation. In this case, convergent orientation is defined as reads with positive orientation when the first sequenced has smallest mapped coordinate (+/+) and negative orientation when the first sequenced read has largest mapped coordinate (-/-).) The remaining discordant fragments indicate potential SVs or sequencing/alignment errors.

Although researchers typically focus on common classes of SVs, such as deletions and inversions, more generally a SV corresponds to a rearrangement creating one or more novel adjacencies between pairs of locations in the reference genome. That is, two locations *a *and *b*, which were originally separated in the reference genome, are now adjacent in the test genome. For example, a deletion creates one novel adjacency while an inversion creates two (Figure 1a). Following the terminology of VCF (Variant Call Format) version 4.1 [45], we refer to locations *a *and *b *individually as breakends and as mated breakends when paired at either end of a SV created by a rearrangement.

We define a predicted SV  as a pair V=(F,B) where $F=\left\{f_{1},f_{2},\ldots ,f_{k}\right\}$ is a set of *k *discordant fragments containing the novel adjacency, and  is the breakend polygon, a region describing all possible mated breakends (*a*,*b*) determined by the discordant fragment mappings (Figure 1b). The breakend polygon is defined by the positions of the mapped ends of each fragment and the minimum ($L_{\text{min}}$) and maximum ($L_{\text{min}}$) length of fragments. If  is a true SV, then there is an ordered pair (a,b)∈B corresponding to a novel adjacency created by the rearrangement. That is, there is a (a,b)∈B such that *a *and *b *are the breakends of the SV in the reference genome. (See Materials and methods and [33] for more information on how the breakend polygon is defined.)

Discordant and concordant fragments provide complementary information about a variant. Discordant fragments define the breakend polygon  while concordant fragments (or lack thereof) provide additional information about the precise location of the breakends within the polygon. If *a *and *b *represent mated breakends created by a deletion, inversion or other rearrangement in the reference genome, we should see a decrease in the coverage by concordant fragments at these points. The type of signal we expect to see depends on the type of SV present (Figure 1c). For a deletion, we expect a drop in the coverage of concordant fragments throughout the genomic interval [*a*,*b*]. This is commonly known as the RD signal and has previously been exploited to reveal copy number variants [38]. For an inversion or reciprocal translocation, we expect a sharp drop in coverage in the regions immediately surrounding *a *and *b *as many of the fragments containing *a *or *b *in the test genome are discordant when mapped to the reference. However, there is no drop in coverage 'inside' the inversion or translocation. We define this highly local drop in coverage as the breakend read depth (beRD) signal.

We develop a probabilistic model based upon the mapped locations of all fragments, concordant and discordant, in the test genome. By doing so we integrate both the presence of discordant fragments (PR signal) and concordant coverage (RD signal) into a single probabilistic method, GASVPro. In addition, GASVPro directly estimates the location of the breakends *a *and *b *for a SV  and classifies the prediction as homozygous or heterozygous. We first present our model in the restricted context where every fragment has a unique mapping to the reference genome. Then, we extend our model to fragments with multiple alignments by using an MCMC approach to sample over the possible mappings for each fragment.

#### Probability of a structural variant

We determine the probability of a potential SV  by considering the number, *k*, of discordant fragments as well as the beRD, the depth of coverage at each candidate breakend. By doing so, we directly estimate the novel adjacency created by  by considering all possible mated breakends consistent with the discordant fragments. Since our formulation depends only on the process of sampling fragments from the test genome, and not on the class of variant, our probabilistic model is applicable to generic rearrangements.

We follow the Langer-Waterman model [46] of sequencing and assume that the starting positions of the fragments are sampled from the test genome uniformly so that the left positions of fragments follow a Poisson process with parameter λ. If all sequenced fragments had fixed length *L*, the number of fragments containing an arbitrary point *p *from the test genome, called the coverage of *p*, would simply be the number of fragments sampled with left endpoint in the interval [*p *- *L *+ 1,*p*]. According to the Poisson process, the coverage of a point *p *follows a Poisson distribution with mean λ*L*. In general, we do not know the size of any particular fragment and thus we use the average fragment length, $L_{avg}$, and model the coverage of *p *by a Poisson distribution with mean $\lambda_{c}=\lambda L_{avg}$.

If *p *is sufficiently far from all sites of structural variation, we expect all sequenced fragments containing *p *to be concordant with respect to the reference genome. However, if *p *is the breakend of an SV, coverage will be reduced, as there will be fewer concordant fragments containing the breakend. In particular, the distribution of the number of fragments containing a breakend *p *is approximated by a Poisson distribution with mean $\lambda_{d}=\left(L_{avg}-2\times readlength\right)\lambda$ (see Materials and methods and Figure A1 in Additional file 1).

Consider a candidate SV V=(F,B). If  is a true SV, then there is an ordered pair, (a,b)∈B, corresponding to a novel adjacency in the test genome created by the rearrangement. As such, the number of concordant fragments containing *a *or *b *should be lower than expected for an arbitrary point in the reference genome. Alternatively, if  is not a true SV, then the coverage of points *a *and *b *by concordant fragments will follow the Poisson distribution with mean $\lambda_{c}$. We next describe the probability of a variant  by conditioning on the choice of breakends and number of copies of the novel adjacency (*a*,*b*) in the test genome. Specifically, for a candidate novel adjacency (a,b)∈B, let *C*(*a*,*b*) = {0,1,2} indicate the number of copies of the novel adjacency in the test genome. (Here we are considering only copy-neutral or copy number loss events (for example, deletions) and not duplications. The extension to the latter case is future work.) We consider three events: (1) *a *and *b *are breakends of a homozygous SV, (*C*(*a*,*b*) = 2); (2) *a *and *b *are breakends of a heterozygous SV (*C*(*a*,*b*) = 1); (3) *a *and *b *are not SV breakends (*C*(*a*,*b*) = 0).

For a candidate breakend *p*, we define the breakend read depth (beRD), *n*(*p*), to be the number of mapped fragments containing *p*. In the case that *a *and *b *are endpoints of a homozygous SV, we expect *n*(*a*) = *n*(*b*) = 0; that is, any concordant fragment containing *a *or *b *represents a mapping error. We assume that mapping errors are independent and the probability, $p_{err}$, of an erroneous mapping is the same for all fragments. In addition, the number, *k*, of discordant fragments in  is drawn from a Poisson distribution with parameter $\lambda_{d}$. Thus, conditional on a choice of breakends (*a*,*b*), the probability that  represents a homozygous SV (that is, *C*(*a*,*b*) = 2) is given by:

(1) $$P\left(V|C\left(a,b\right)=2\right)=\left(p_{err}^{n\left(a\right)+n\left(b\right)}\right)Pois\left(\lambda_{d};k\right)$$

where $Pois\left(\lambda ;k\right)=\lambda^{k}\text{exp}\left(-\lambda \right)/k!$ is the probability density function for the Poisson distribution with mean *λ*. One could explicitly define the unconditional probability that  is a homozygous variant by examining the likelihood that each pair (a,b)∈Bare the true mated breakends. Instead, we make a simplification by taking the maximum probability over all possible breakend pairs:

(2) $$P\left(V|C\left(B\right)=2\right)=\underset{\left(a,b\right)\in B}{\text{max}}P\left(V|C\left(a,b\right)=2\right)$$

where by *C*(*B*) = 2 we mean the breakpoint region  defines a homozygous SV.

Similarly, if (a,b)∈B are mated breakends of a heterozygous variant, *C*(*a*,*b*) = 1, we expect the number of concordant fragments that contain *a *or *b *to follow a Poisson distribution with mean $\lambda_{c}/2$ and the number of discordant fragments that contain the novel adjacency (*a*,*b*) to follow a Poisson distribution with mean $\lambda_{c}/2$, respectively. Thus, conditional on the choice of breakends (*a*,*b*), the probability that *V *represents a heterozygous SV is given by:

(3) $$P\left(V|C\left(a,b\right)=1\right)=Pois\left(\frac{\lambda_{c}}{2};n\left(a\right)\right)Pois\left(\frac{\lambda_{c}}{2};n\left(b\right)\right)Pois\left(\frac{\lambda_{d}}{2};k\right)$$

As before, we define the unconditional probability that  represents a heterozygous variant by:

(4) $$P\left(V|C\left(B\right)=1\right)=\underset{\left(a,b\right)\in B}{\text{max}}P\left(V|C\left(a,b\right)=1\right)$$

Finally, if *a *and *b*, (a,b)∈B, are not breakends of a SV, *C*(*a*,*b*) = 0, we expect the number of concordant fragments containing the breakpoints *n*(*a*) and *n*(*b*) to follow Poisson distributions with mean λc and all *k *discordant fragments to be mapping errors, each occurring independently with probability $p_{err}$. Thus, conditional on a choice of (*a*,*b*), the probability that  does not represent a SV is given by:

(5) $$P\left(V|C\left(a,b\right)=0\right)=Pois\left(\lambda_{c};n\left(a\right)\right)Pois\left(\lambda_{c};n\left(b\right)\right){p_{err}}^{k}$$

As before, we define the unconditional probability that  is not a variant by:

(6) $$P\left(V|C\left(B\right)=0\right)=\underset{\left(a,b\right)\in B}{\text{max}}P\left(V|C\left(a,b\right)=0\right)$$

For each candidate variant we decide between alternatives using a likelihood ratio. That is, we compare the probability that  represents a SV (homozygous or heterozygous) with the probability that  is an error as follows:

(7) $$\Lambda \left(V\right)=\underset{\left(a,b\right)\in B}{\text{max}}\frac{\text{max}\left\{P\left(V|C\left(a,b\right)=2\right),P\left(V|C\left(a,b\right)=1\right)\right\}}{P\left(V|C\left(a,b\right)\right)=0}$$

In practice we report variants  where logΛ(V) exceeds a prescribed threshold. In addition to assigning a likelihood to a SV, our formulation determines a maximum likelihood estimate for the novel adjacency (*a*,*b*) and if a variant is homozygous or heterozygous.

#### Probability of a deletion

The model in the previous section presented considers only coverage at the breakends *a *and *b*. However, deletions have a stronger signal of reduced coverage, as shown in Figure 1c. That is, for a true deletion coverage by concordant fragments should be reduced throughout the entire deleted segment. Let V=(F,B) be a predicted deletion supported by *k *discordant fragments and define $a_{max}=\underset{a}{\text{arg}\text{max}}\left\{\left(a,b\right)\in B\right\}$ and $b_{\text{min}}=\underset{b}{\text{arg}\text{min}}\left\{\left(a,b\right)\in B\right\}$. Then, for any choice of mated breakends (a,b)∈B, the interval $I\left(B\right)=\left[a_{max},b_{min}\right]$ must be deleted. As before, we expect the number *n*(*I*) of concordant fragments whose mappings overlap the interval *I*(*B*) to be Poisson distributed with mean:

$$\lambda_{I}=\lambda \left(\left(b_{min}-a_{max}\right)+L_{avg}\right)$$

Let *C*(*B*) = {0,1,2} be the number of copies of the variant in the test genome. We consider the probability of three events:

$$\begin{array}{c} P\left(V|C\left(B\right)=2\right)=\left(p_{err}^{n\left(I\right)}\right)Pois\left(\lambda_{d};k\right)\\ P\left(V|C\left(B\right)=1\right)=Pois\left(\lambda_{I}/2;n\left(I\right)\right)Pois\left(\lambda_{d}/2;k\right)\\ P\left(V|C\left(B\right)=0\right)=Pois\left(\lambda_{I};n\left(I\right)\right)\left(p_{err}^{k}\right) \end{array}$$

and finally the likelihood of a deletion compared to a mapping error:

(8) $$\Lambda \left(V\right)=\frac{\text{max}\left\{P\left(V|C\left(B\right)=2\right),P\left(V|C\left(B\right)=1\right)\right\}}{P\left(V|C\left(B\right)=0\right)}$$

There are several additional factors we consider when using our model on sequencing data. First, there are factors other than SVs that can impact the coverage of concordant fragments over an interval. As such, to adjust for differences in the ability to map reads throughout the genome, in our model for deletions we scale the number of concordant fragments by the local mapability of the putative deleted interval. Second, since in this study we are primarily interested in inversion and deletion SVs, in practice we utilize a heuristic to eliminate regions of the genome with extremely high coverage by concordant fragments. Further information on these practical details are given in the Materials and methods section.

#### Selecting a mapping for each fragment

In the previous sections, we assumed that there was a single high-quality alignment for all reads and therefore one high-quality alignment for each fragment. However, some reads may have multiple high-quality alignments due to repetitive sequences in the reference or sequencing errors in the reads. Selecting one of the possible alignments for each read from the pair defines an alignment of the fragment. Since each fragment represents a unique contiguous region of the test genome, at most one alignment is the correct one and we refer to this as the mapping of the fragment.

Selecting a mapping for each fragment defines the set of concordant and discordant fragments and an associated set of SVs that could be evaluated using the model in the previous section. Although any such selection defines a fragment configuration consistent with the data, each selection has a different probability. Thus, rather than selecting a mapping for each fragment in advance, we consider the space of all possible mappings for all fragments and use a MCMC approach to sample from the space of possible mappings in proportion to their probability.

With these distinctions, we now revisit our notions of 'concordant' and 'discordant' from above. A concordant fragment is a fragment whose unique mapping is concordant. That is, both reads have a single high-quality alignment to the reference and the alignments are concordant with respect to the sequencing process. A discordant fragment is a fragment whose entire set of alignments are discordant. (Note, this formulation ignores any fragment with multiple alignments, at least one of which is concordant.)

Let $F=\left\{f_{1},f_{2},\ldots ,f_{m}\right\}$ be the set of all discordant fragments. Suppose that the two reads from a fragment f∈F map to *s *and *t *locations, respectively. An alignment of a fragment corresponds to selecting an alignment for each read, and thus we define $A\left(f\right)=\left\{\left(x_{i},y_{j}\right)\right\}$ where *i *= 1,2,...*s *and *j *= 1,2,...*t *as the set of all alignments for a fragment , only one of which may be the true mapping. Let $A=\left\{\left(A(f_{1}\right),A\left(f_{2}\right),\ldots ,A\left(f_{m}\right)\right\}$ be the set of alignments for all fragments.

**Figure 2:**
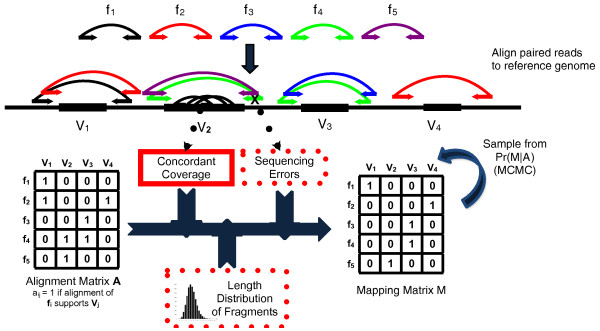
**Overview of the GASVPro**. Fragments  from a test genome are sequenced and the resulting paired reads are aligned to the reference. A fragment may either have a unique mapping or be ambiguous with multiple alignments to the reference. Following clustering of alignments (with GASV), the set  of possible structural variants and the fragments whose alignments support these variants are recorded in the alignment matrix *A*. As each fragment originates from a single location in the test genome, a fragment supports at most one structural variant. Thus, the mapping matrix *M *records the 'true' mapping for each fragment. GASVPro scores mapping matrices according to a generative probabilistic model that incorporates concordant mappings. GASVPro utilizes an MCMC procedure to efficiently sample over the space of possible mapping matrices defined by the alignment matrix *A*. The underlying probabilistic model can be easily generalized to consider additional features indicative of a 'true' mapping, such as the empirical fragment length distribution or probability of sequencing errors.

Let $V=\left\{V_{1},V_{2},\ldots ,V_{n}\right\}$ be a set of candidate SVs supported by , as before $V_{i}=\left(F_{i},B_{i}\right)$.  is computed by clustering discordant pairs that support the same variant. (In the results below, we use GASV [33] to obtain the breakpoint polygon associated with each $V_{i}$; however, this step could be replaced by a different clustering method.) We represent the set of all possible SVs supported by  with an *m*×*n *binary (0-1 valued) *alignment matrix*, $A=\left[a_{ij}\right]$, with rows corresponding to fragments $\left\{f_{1},f_{2},\ldots ,f_{m}\right\}$ and columns corresponding to possible SVs $\left\{V_{1},V_{2},\ldots ,V_{n}\right\}$. Here $a_{ij}=1$ if fragment $f_{i}$ supports SV $V_{j}$ (that is, there is an element of $A\left(f_{i}\right)$ that supports variant $V_{j}$ and thus $f_{i}\in F_{j}$) and $a_{ij}=0$ otherwise (Figure 2).

We assume that a discordant fragment supports at most one SV. Thus, our goal is to select the single 'correct' mapping for each fragment, according to some criterion. Such a selection corresponds to a binary m×n mapping matrix $M=\left[m_{ij}\right]$, where $m_{ij}=1$ if fragment $f_{i}$ is assigned to SV $V_{j}$.  satisfies the following:

1. $m_{ij}\leq a_{ij}$; that is, $m_{ij}=1$ only if $a_{ij}=1$,

2. $\underset{i}{\sum }m_{ij}\leq 1$for all *i*; that is, each row in  has at most one non-zero entry.

Finally, as before, the probability of variants depends on the associated copy number, C(B), of a variant. We explicitly distinguish between homozygous and heterozygous SVs by including a binary vector $C=\left(C_{1},C_{2},\ldots ,C_{n}\right)$ where $C_{j}=C\left(B_{j}\right)$. If any discordant fragments are assigned to $V_{j}$, we require $C_{j}>0$. Together  and  define the differences between the test and reference genome.

#### Probability of a mapping matrix

Our data *D *consists of a set  of discordant fragments, a set  of alignments, a set  of possible SVs, and the positions of all concordant mappings in the genome. We next generalize our probability model from the previous section to the probability of a mapping matrix based on the generation of the data *D *from a given genome.

For a mapping matrix  and discordant fragment $f_{i}$, let $\gamma_{i}\left(M\right)$ denote the column index of the 1 in the *i*-th row, or 0 if $f_{i}$ is not assigned. For a mapping matrix  and a variant , let $R_{j}\left(M\right)$ be the set of rows with a 1 in column *j*. The support, $S_{j}\left(M\right)$, of variant *j *is defined as the number of assigned discordant fragments:

$$S_{j}\left(M\right)=\;\left|R_{j}\left(M\right)\right|=\underset{i}{\sum }m_{ij}$$

Finally, we define the total number of variants V(M) predicted by :

$$V\left(M\right)=\;\left|\left\{j:S_{j}\left(M\right)>0\right\}\right|.$$

Given an alignment matrix , the probability of a mapping matrix  is a function of the number of fragments supporting each variant with positive support. We assume that the number of variants with positive support follows an exponential distribution with parameter . Finally, if a discordant fragment is assigned to none of the SVs, then this fragment represents a mapping error, an event with probability $p_{err}$. Thus, we have:

(9) $$P\left(M,C|A\right)\propto \eta e^{-\eta V\left(M\right)}\underset{j:S_{j}\left(M\right)>0}{\prod }P\left(V_{j}\left(M\right)|C_{j}\left(M\right)\right)\underset{i:\gamma_{i}\left(M\right)=0}{\prod }p_{err}$$

where ${\text{V}}_{j}\text{(M) = (}F_{j}\left(M\right),B_{j}\left(M\right))$ is the SV in column *j *supported by fragments $F_{j}\left(M\right)$, corresponding breakpoint region $B_{j}\left(M\right)$ and $C_{j}\left(M\right)=C\left(B_{j}\left(M\right)\right)$. As above, we utilize a different model for predicting deletions that also includes read depth inside the putative deleted interval. Finally, we define P(M|A) by defining  by selecting the most likely copy number $C_{j}$ for each *j*:

(10) $$P\left(M|A\right)=\underset{C}{\text{max}}P\left(M,C|A\right).$$

Note that  specifies a unique mapping for each fragment supporting a variant; thus, one solution would be to consider P(M|A) over all possible mapping matrices. However, because the number of possible mapping matrices  grows exponentially with the number of fragments, we use a MCMC procedure to efficiently sample from the space possible mapping matrices  (Figure 2; Section A2 and Figures A2, A3 in Additional file 1). Our MCMC procedure converges to the unique stationary distribution given in Equation 10.

**Figure 3:**
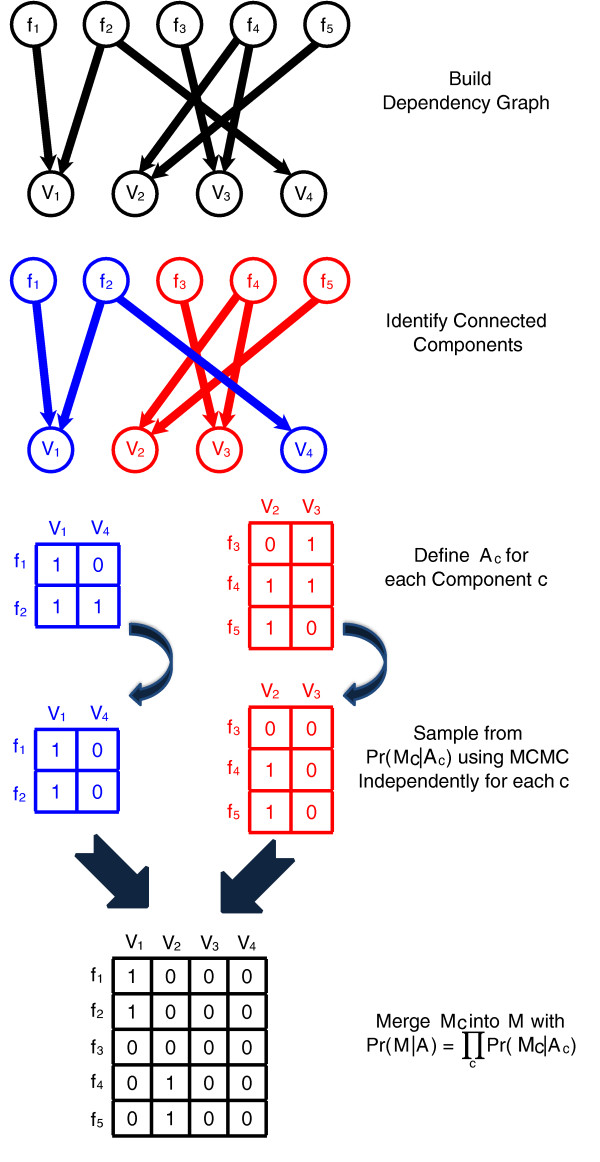
**Sampling over connected components of alignments**. The probabilistic model *P*(*M*|*A*) used by GASVPro allows for efficient decomposition of the original space of mapping matrices *M *into independent components. Thus, we sample using MCMC on each component independently and merge the results.

Although the space of mapping matrices has high dimension, our MCMC procedure remains computationally tractable because our sampling procedure may be performed on disjoint sets of fragment mappings and the variants they support. Thus, our MCMC samples independently on each such component and the combination of these samples converges to the same stationary distribution as sampling over the complete space. See Figure 3 for a schematic. In the Materials and methods section, we provide a complete description of our MCMC sampling procedure and provide further discussion in Additional file 1.

#### Deriving the predicted structural variants

Our MCMC procedure samples mapping matrices in proportion to their probability P(M|A); however, our ultimate goal is to report a final set of SV predictions. One approach to SV prediction is to select a single  according to some criteria; for example, the  that minimizes the total number of SVs predicted. This approach is used by a number of SV detection methods that consider multiple assignments for fragments, such as VariationHunter [42] and Hydra [34]. We instead predict SVs by considering the entire space of mapping matrices  according to P(M|A) as described in the Materials and methods. In practice, we found only minor differences in the receiver operating characteristic (ROC) curves for the different reporting methods we considered (Figure A4 in Additional file 1).

### Results on sequenced data

We applied GASVPro to simulated paired-end data on the Venter Genome (HuRef) [47], as well as two previously sequenced human genomes, NA18507 [14] and a European individual, NA12878, from the 1000 Genomes study [44]. We also compared results from GASVPro to two previously published methods, Hydra [34] and BreakDancer [32], as well as the original GASV. (We also performed some comparisons with VariationHunter [42]. Since results were strikingly similar to Hydra, as previously noted in [34], and we were unable to process the full datasets for NA12878 and NA18507 using the current publicly available distribution of VariantionHunter, we present only the results for Hydra.) Finally, we compare to CNVer, a method combining RD and PR to detect copy number variants [38].

These methods, and other similar SV prediction programs, typically employ several steps, including alignment of reads to the reference genome, predicting SVs from alignments, post-processing predictions (for example, pruning a set of predicted SVs to remove redundancy) and comparison to known variants. In an effort to directly compare the performance of the SV prediction algorithms, rather than the specific pre- and post-processing steps, we standardized the alignment, post-processing and comparison steps. In particular, we used the same read alignments for all methods. (Note this involved modifying the source code for Breakdancer to consider only a user-specified set of discordant fragments.) For GASVPro and Hydra, the methods that allow fragments to have multiple possible alignments, we realigned reads to the reference genome with Novoalign [48] and distinguish results on the full set of alignments (GASVPro and Hydra) from results on only the high-quality unique alignments (GASVPro-HQ or Hydra-HQ). Before comparing results, redundant predictions were removed with the same pruning procedure for each method (see Materials and methods).

We compare predictions to a known set of variants using the double uncertainty metric, a novel metric developed to represent uncertainties in the breakpoint locations for both the predictions and the known variants (see Materials and methods; Figures A5 and A6 in Additional file 1). We use a ROC type analysis to show the number of novel predictions and true positives for each method as a function of the number of supporting fragments (Hydra, Breakdancer, GASV), the predicted depth of coverage (CNVer) or the likelihood of a predicted variant (GASVPro). Note that in the results shown below, GASVPro-HQ and GASV consider the same set of high quality unique alignments and utilize the same clustering algorithm. As such, both methods have the same maximum sensitivity, but GASVPro-HQ has higher specificity due to our probabilistic model. On the other hand, GASVPro uses a larger set of alignments, including lower quality and ambiguous alignments, and as such GASVPro can achieve higher sensitivity than GASVPro-HQ and GASV.

#### Simulated data

We first test GASVPro on simulated data generated from the Venter genome [47]. We produced a synthetic dataset by inserting the list of annotated SVs on chromosome 17 of Venter's genome (8,801 deletions, 8,572 insertions and 4 inversions) into the human reference genome (hg18). These SVs varied in length from one to several thousands of bases. We simulated 100× coverage of this chromosome by 50-bp PRs with a mean fragment length of 200 bp and a standard deviation of 20 bp using the SAMtools wgsim program [49]. For all methods, the resulting sets of predictions were pruned and compared to known variants with the double uncertainty metric with reference uncertainty set to 0 (see Materials and methods).

**Figure 4:**
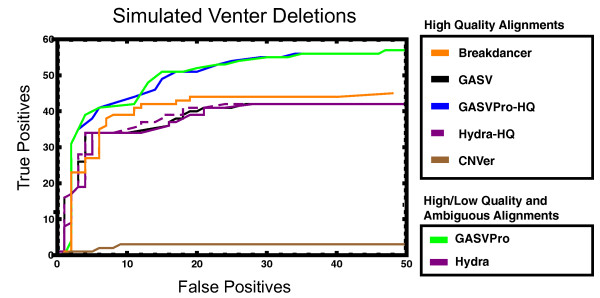
**Simulated Venter chromosome 17**. ROC curves comparing deletion predictions for Breakdancer, GASV, GASVPro-HQ, Hydra-HQ, CNVer, GASVPro and Hydra to the 124 deletions from Venter chromosome 17 with minimum deletion length 125 bp. All methods analyzed the same set of high-quality unique mappings; in addition, GASVPro and Hydra considered a set of lower-quality alignments, including ambiguous fragments with multiple alignments. Predictions of all methods were post-processed in an identical fashion and the resulting predictions were compared to the known coordinates for the Venter deletions according to the double uncertainty metric (see Methods).

The lengths of deletions that are readily predicted from PRs depend on fragment size [11]. To mirror the procedures used on the sequenced genomes, we only considered fragments with mapped length ≥$2\times L_{\text{max}}$ (where $L_{\text{max}}=293$) as potential deletions. We compared predictions from all methods to the 124 deletions with length ≥125 bp. Figure 4 compares all methods on this data set; compared with GASV, Breakdancer and Hydra, GASVPro is over 50% more specific at maximum sensitivity.

All methods had greater sensitivity than CNVer, which made 218 predictions but detected only 3 deletions with the double uncertainty metric. The lower sensitivity of CNVer can be explained in part by internal filtering: the published code of CNVer reports only copy-number events that are larger then 1 kb, which eliminates all but 9 out of 124 simulated deletions. In addition, the reported coordinates from CNVer lie farther from true breakends, although the predicted deletion interval typically contains the true deletion. We note that 16 of 218 CNVer predictions completely contained a true deletion, including 5 of 9 deletions larger than 1 kb. Thus, some of the difficulties with CNVer result from how it merges potential copy-number variants before reporting a final set of predictions (Section A3 in Additional file 1).

We next discuss GASV compared with Breakdancer, Hydra and Hydra-HQ. Before removing redundant predictions by pruning, GASV predicts 648 deletions with at least one supporting fragment, which detects 60 Venter deletions. Thus, the maximum sensitivity is 48%. A common method to increase specificity is to increase the minimum number of supporting fragments for a prediction. As discussed previously, however, many predictions from SV methods overlap. Removing these overlapping predictions (see Materials and methods) improves performance more than increasing the number of supporting fragments. For GASV, restricting the set of predictions to those with at least two supporting fragments results in 244 predictions but detects only 46 deletions. In comparison, pruning the 648 predicted deletions with at least one fragment retains 347 predictions that detect 57 true deletions. In comparison, Hydra-HQ and Hydra had slightly lower sensitivity, predicting only 44 deletions at maximum sensitivity, but had similar overall performance to GASV. Breakdancer had similar performance throughout with slightly higher sensitivity than Hydra/Hydra-HQ and GASV and equal specificity.

The integrative probabilistic model used by GASVPro greatly improves specificity. Analyzing only high quality unique mappings, GASVPro-HQ predicts only 64 deletions with positive log likelihood, logΛ(V)> 0, which include 50 true deletions. Note that these 64 predictions are a subset of those predicted by GASV. Thus, compared to GASV, GASVPro-HQ has a substantially lower false positive rate at highest sensitivity. The improved specificity of GASVPro-HQ over GASV is evidence that our likelihood statistic is a better predictor of true variants than the number of supporting fragments (see also Figure A7 in Additional file 1 for a comparison). Including low-quality and ambiguous alignments increases the space of possible variants substantially without significantly increasing the number of detectable deletions. That is, the full set of possible alignments suggest 1,051 potential deletion events that overlap, at most, 61 out of 124 true deletions. However, GASVPro has similar performance to GASVPro-HQ throughout. This suggests that the MCMC sampling method is able to successfully eliminate many false positive predictions even with a much larger number of initially possible variants.

Finally, we compared the ability of all methods to identify the four inversions on Venter chromosome 17 (Table 1). On this simulated data our probabilistic formulation and MCMC sampling method proved beneficial. GASVPro-HQ identified three inversions with four predictions while GASVPro identified all four inversions with no false positive predictions. Notably, the additional inversion identified by GASVPro had breakends within a segmental duplication. In this case a total of 170 fragments had two possible alignments, each of which corresponded to a potential inversion SV, but only one of which is the true inversion. The beRD signal used by GASVPro allowed the algorithm to successfully distinguish between the true and false prediction. The MCMC algorithm used by GASVPro assigned a greater likelihood to the true prediction because 23 concordant fragments map to the breakend polygon for the false prediction. In comparison, Hydra requires ten predictions to detect all four inversions. GASV and Breakdancer are slightly less sensitive, detecting only three quarters of known inversions. Thus, GASVPro is the only method to attain optimal sensitivity and specificity on the inversion data set.

**Table 1:** Comparison of performance of methods with respect to identifying the four inversions on Venter chromosome 17

Method	Minimum number to detect 3	Minimum number to detect 4
**High quality alignments**		
Breakdancer	3	NA
GASV	3	NA
GASVPro-HQ	3	NA
Hydra-HQ	4	NA
		
**All alignments**		
GASVPro	3	4
Hydra	5	10

GASVPro is the only method with perfect specificity and sensitivity, detecting all four inversions with no false positive predictions. NA, not applicable.

#### Sequencing data

##### NA12878 deletions

We next compared the methods on Illumina sequencing data of a CEU individual, NA12878, from the 1000 Genomes Project. There are two sets of validated SVs available for this individual. First, deletions and inversions were validated from a previously published fosmid study [16] and deletions were separately validated as part of the 1000 Genomes Project [44]. In addition, the validated deletions from the 1000 Genomes data set were also annotated as homozygous or heterozygous.

Individual NA12878 was sequenced in both Pilot 1 (≈4× coverage) and Pilot 2 (≈40× coverage) of the 1000 Genomes Project. For Pilot 1, a single library was sequenced with a read length of 37 bp and an average fragment size of 230 bp. For Pilot 2, multiple libraries were sequenced with read lengths from 37 to 52 bp and an average fragment size of 150 to 350 bp. Thus, we analyzed both datasets to examine the effect of different coverage on the ability of methods to predict SVs.

**Figure 5:**
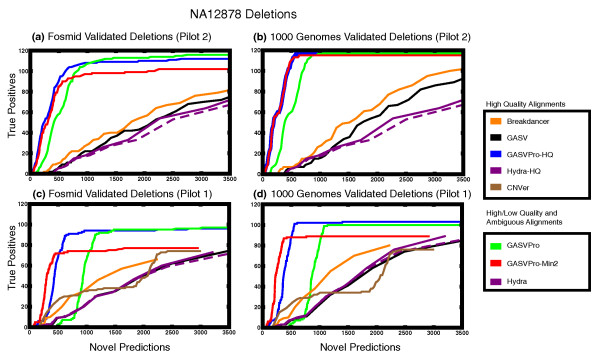
**An 'ROC curve' comparing the number of known deletions that were correctly predicted (true positives) and the number of novel deletion predictions using sequencing data and validated deletions from the individual NA12878 in Pilot 1 and Pilot 2 of the 1000 Genomes Project**. (As discussed in the Methods section, we separately considered two sets of validated deletions [16,44].)

In Figure 5 we plot 'ROC curves' comparing the predictions of GASV, GASVPro, GASVPro-HQ, Hydra, Hydra-HQ, CNVer and Breakdancer on data from Pilot 2 (Figure 5a,b) and Pilot 1 (Figure 5c,d) to both sets of validated deletions. Since CNVer could only be run on a single library, we consider CNVer results on Pilot 1 data alone. Because the complete list of true SVs in the genome is not yet known, we cannot compute the number of false positives/negatives. Thus, we plot the number of novel predictions compared to true positives. We also considered only predictions with at least two supporting fragments and plot these results as GASVPro-Min2. As before, to assess the difference due to low quality and ambiguous mappings, we plot both Hydra and Hydra-HQ; the latter is Hydra run on only high-quality uniquely mapped fragments.

We first consider the results on the higher coverage Pilot 2 data (Figure 5a,b). Four curves represent methods run on only uniquely mapped fragments: GASV, GASVPro-HQ, Hydra-HQ and Breakdancer. Breakdancer has slightly improved performance compared to Hydra-HQ and GASV, attaining equal sensitivity with up to 200 fewer predictions throughout. However, this may be an artifact of Breakdancer's aggressive clustering procedure (discussed in Section A3 of Additional file 1). GASVPro-HQ has the best overall performance with over a 85% reduction in novel predictions at highest sensitivity compared to Breakdancer, GASV and Hydra.

Of the three methods that use all alignments (GASVPro, GASVPro-Min2 and Hydra), GASVPro has the highest sensitivity, detecting 119 of 139 true deletions with 19,715 novel predictions on the set of validated deletions from the 1000 Genomes study. By increasing the minimum likelihood threshold, and thus reducing the number of predictions, GASVPro predicts 114 of 139 true deletions with only 907 novel predictions; this represents a 95% decrease in the number of novel predictions with only a 3% decrease in true positives. GASVPro-Min2 has higher specificity than GASVPro, making around 200 fewer predictions than GASVPro at equal sensitivity. Notice the addition of ambiguous mappings alone does not greatly improve performance as the behavior of Hydra and Hydra-HQ is very similar, with Hydra being slightly more sensitive. Thus, regardless of whether unique or ambiguous fragments are used, combining both read depth and PRs with our probabilistic model (GASVPro-HQ, GASVPro-Min2 or GASVPro) results in significant improvements to sensitivity and specificity.

In addition to improving the ability to successfully predict true deletions, our probabilistic model also accurately classifies these variants as homozygous or heterozygous. GASVPro-HQ correctly classified 104 out of the 119 known deletions with highest likelihood as homozygous or heterozygous according to the annotations in the 1000 Genomes data set. Remarkably, all 28 homozygous variants in this set were correctly classified even though some had fewer supporting discordant fragments than many correctly classified heterozygous variants.

On Pilot 1 data, we also compare the performance of CNVer, which uses both discordant mappings and read depth to predict copy number variants. In contrast to the simulated data set above, all known deletions analyzed here are larger than 1 kb and thus CNVer attains similar sensitivity to PR methods, like Hydra, GASV and Breakdancer. However, the number of discordant fragments per prediction, the criteria used to rank results for PR methods, provides a better trade off between true and false positive predictions than the estimated depth of coverage, which we use to rank CNVer predictions.

Even with the reduced coverage, compared to Pilot 2, the benefits of our probabilistic models are evident, and GASVPro outperforms all competing methods. GASVPro-HQ and GASVPro-Min2 have improved performance compared to Hydra, Hydra-HQ, Breakdancer and GASV. Note that the specificity for GASVPro drops below all other methods at the highest likelihood threshold (Figure 5c,d). This drop in performance is due to many predictions of GASVPro consisting of only a single discordant fragment mapping to a large region with very few concordant fragments. While it is possible these are true variants, it is more likely that most of them are false positives and, as such, eliminating these predictions (GASVPro-Min2) restores performance to that obtained by GASVPro-HQ. On this dataset, GASVPro-HQ correctly classifies 84 out of the 102 known deletions with highest likelihood as homozygous or heterozygous. As in the Pilot 2 data set, all 26 of 102 homozygous deletions were correctly classified, 3 of which have fewer than 3 supporting fragments.

We next evaluate the effect of increased coverage on each method by comparing the results from Pilot 2 (Figure 5a,b) with Pilot 1 (Figure 5c,d). For the methods utilizing only discordant mappings (Hydra, Hydra-HQ, GASV, and Breakdancer) performance is similar between Pilot 1 and Pilot 2 data. In contrast, performance of our probabilistic methods, GASVPro-HQ, GASVPro-Min2 and GASVPro, increases substantially with coverage. The maximum sensitivity of GASV Pro and GASVPro-HQ increases by about 20% on both data sets, from 97 to 119 and 96 to 114, respectively, for the fosmid validated set and 100 to 119 and 103 to 120 on the 1000 Genomes validated set. This improved performance results from integration of both discordant fragments (PR signal) and concordant fragments (RD signal). Increasing the sequencing coverage increases both discordant and concordant mappings throughout the genome. However, higher discordant coverage contributes to both true and false predictions, and thus methods that analyze only discordant fragments are less able to leverage the increased coverage to distinguish true from false predictions. In contrast, increased coverage by concordant fragments leads to sharper delineations between normal and deleted regions in the genome. Although it is possible that CNVer results would have also improved with the higher coverage data, a comparison was not possible as multiple libraries are not supported in the published CNVer implementation.

Finally, we remark on a practical difficulty in assessing the performance of methods on sequenced genomes. As indicated above, the complete set of SVs on these genomes is unknown. Thus, it is possible that predictions classified as 'novel predictions' could in fact be true, but yet unknown, variants. In addition, the set of validated variants that we use as true positives may not be representative of all SVs in these genomes. For example, we attained significant improvements in specificity for both inversions and deletions on NA12878 when we used a 'homozygous-only" model in GASVPro (Figure A8 in Additional file 1). This suggests that the set of known variants may underrepresent heterozygous deletions and inversions, which are presumably more difficult to detect and validate.

##### NA18507 deletions

**Figure 6:**
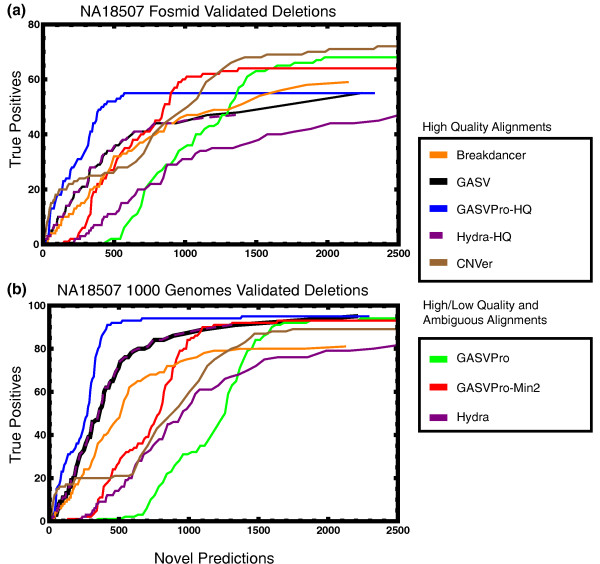
**An 'ROC curve' comparing the number of known deletions and novel deletion predictions for NA18507**. (As for individual NA12878, we separately considered two sets of validated deletions [16,44].)

We next compare all methods on previously published Illumina data [14] for the YRI individual NA18507. This genome was sequenced to high coverage (35 bp reads, ≈200 bp fragment length, 30× coverage) and, as for NA12878, there were two available validated sets of deletions and one set of inversions. In Figure 6, we show the results for previously validated fosmid deletions (Figure 6a) and validated deletions from the 1000 Genomes Project (Figure 6b). Since CNVer published their predictions on this data set, we compare directly to their previously reported results.

As above, employing our integrative probabilistic model for discordant fragments with unique mappings, GASVPro-HQ greatly improves performance compared to the original GASV. Using GASV alone, at maximum sensitivity we predict 55 of 93 deletions from the fosmid study with 2,240 novel predictions. In comparison, GASVPro-HQ successfully predicts the same 55 of 93 deletions with only 573 novel predictions. Similarly, for the 1000 Genomes deletions, at maximum sensitivity GASV predicts 95 of 118 deletions with 2,201 novel predictions while GASVPro-HQ attains the same sensitivity with only 1,372 novel predictions. Thus, using our probabilistic framework provides a two-fold increase in specificity at equal sensitivity. On the fosmid validated deletions, CNVer attains higher sensitivity than other methods and has overall higher specificity than GASV or Hydra at equal sensitivity (Figure 6a). However, this performance is not maintained on both sets of validated deletions (Figure 6b).

Overall, methods that analyze only unique mappings (Breakdancer, GASV, GASVPro-HQ, Hydra-HQ) outperformed those considering lower quality and ambiguous mappings. For this data set, including the full set of mappings (GASVPro and Hydra) greatly increases the number of predictions while, at best, modestly increasing the number of validated deletions that are correctly predicted. Indeed, running Hydra on only the unique mappings yields an 'ROC curve' similar to GASV alone. Although both GASVPro and original GASV match 70 of 93 variants from the fosmid study and 95 of 118 from 1000 Genomes Project, this is at the expense of predicting thousands of novel deletions on each data set, 5,535 and 21,523, respectively. We attain improved performance on the ambiguous data set by considering predictions with more than one supporting fragment, GASVPro-Min2; however, these results are still worse than GASV alone.

The decreased performance of GASVPro and Hydra on this data set, compared to NA12878 above, cannot be solely attributed to the read length as in both cases the sequenced reads were, on average, the same length, 37 bp. The differences seem likely due to difficulties in mapping uniquely to the reference. For NA12878, 31% of all mappings were unique while for NA18507, less than 1.5% of mappings were. In addition, there were more discordant fragments considered for NA18507, but fewer validated SVs. This combination may explain the substantial increase in 'novel' predictions, as compared to known deletions.

##### Inversions

In comparison to deletions, inversion SVs are more difficult to analyze for three reasons. First, there is no difference in read depth across the inversion, but only a change in read depth at the break ends (break end read depth). Second, there are few known inversion variants available for testing. Indeed, the 1000 Genomes SV paper [43] reports thousands of deletions but no inversions. Third, inversion SVs are known to have breakpoints with segmental duplications or other repetitive sequences, and aligning reads to these regions is complicated.

**Figure 7:**
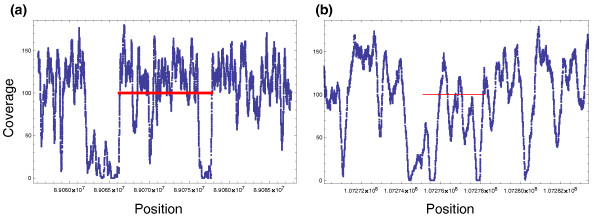
**Concordant coverage per position for two known inversions successfully predicted by GASVPro**. **(a) **A prediction with 99 discordant fragments overlaps a chromosome 4 inversion with left breakend uncertainty of 89.040 to 89.069 Mb and right breakend uncertainty of 89.075 to 89.108 Mb. **(b) **A prediction with 15 discordant fragments overlaps a chromosome 6 inversion with left breakend uncertainty of 107.245 to 107.283 Mb and right breakend uncertainty of 107.277 to 107.315 Mb. For both predictions, a thick red line indicates the minimum and maximum mapped ends (*x*,*y*) for all supporting discordant fragments.

Even with these limitations we demonstrate the benefit of beRD in improving inversion prediction. As noted previously, on the simulated data set the beRD signal allowed GASVPro to correctly assign fragments to the true prediction when there were two choices possible. We now illustrate the beRD signal is beneficial on the real data. In Figure 7, we show the beRD for two inversions identified in NA18507 by GASVPro-HQ. As expected, in both cases there is a noticeable drop in coverage near the potential breakends, demonstrating the benefit of a model that utilizes beRD in addition to discordant fragments.

We compared predicted inversions for all methods to a set of validated inversions from a previous fosmid study [16] (see Materials and methods). The number of validated inversions is significantly smaller than the number of validated deletions; 23 inversions were validated in NA12878 and 10 in NA18507. All methods were far less sensitive in identifying inversions than deletions; maximum sensitivity over all methods was less than 20% on NA12878 and 70% on NA18507.

For all methods, we show the minimum number of inversion predictions needed to identify 1, 2 and 3 out of 23 inversions for NA12878 Pilot 1 and Pilot 2 data (Table 2). On Pilot 1 data our probabilistic models GASVPro and GASVPro-HQ attained improved sensitivity compared to GASV when detecting one and two inversions. In the case of the first inversion, the specificity increased by over 50% for GASVPro and over 80% for GASVPro-HQ. In almost all cases the higher coverage from Pilot 2 improved performance as the same number of inversions are detectable with fewer predictions. However, unlike for deletions, our probabilistic models do not always attain highest specificity. Over all methods, GASV was able to detect 2 inversions with the minimum number of predictions, while GASVPro-HQ detected 1 and 3 inversions with the minimum number of predictions. Finally, including lower quality mappings on this dataset did not yield improved performance; although GASVPro was able to attain highest sensitivity, detecting 4 of 23 inversions, this came at the price of thousands of more predictions.

**Table 2:** Inversion prediction in individual NA12878

Method	Minimum number to detect 1	Minimum number to detect 2	Minimum number to detect 3
**High quality alignments**			
Breakdancer	47 (37)	80 (221)	NA (NA)
GASV	34 (158)	76 (298)	5,028 (NA)
GASVPro-HQ	11 (20)	116 (102)	206 (346)
Hydra-HQ	61 (139)	108 (246)	284 (NA)
			
**All alignments**			
GASVPro	28 (59)	394 (286)	550 (504)
GASVPro-Min2	28 (59)	160 (334)	NA (NA)
Hydra	159 (258)	NA (470)	NA (NA)

We report the results on both Pilot 2 and Pilot 1, with the Pilot 1 results in parentheses. In most cases the sensitivity of inversion detection increases with coverage with more methods correctly predicting three inversions in the higher coverage Pilot 2 data. In some cases, true inversions identified by the uniquely mapped data are lost with the addition of ambiguous alignments. These alignments result in substantially more predictions, which can cause true inversions to be eliminated in the pruning process. The benefit of our probabilistic method and inclusion of the beRD signal is evident as higher specificity is attained by GASVPro and GASVPro-HQ compared to GASV, when predicting the top inversion. NA, not applicable.

Lastly, we analyze inversion results for NA18507 (Table 3). A total of two out of ten inversions are predicted from unique discordant mappings alone. All methods are able to predict these inversions, but Hydra-HQ is able to do so with only 43 predictions, the minimum number across all methods. As in the simulated Venter data, a third true inversion is detected with the inclusion of ambiguous mappings. In this case, GASVPro and GASVPro-Min2 detect three of ten inversions with 60% fewer predictions than Hydra. Thus, while the probabilistic model used by GASVPro is beneficial in some cases, unlike for deletion variants, it does not result in improved specificity for all cases.

**Table 3:** Inversion prediction in individual NA18507

Method	Minimum number to detect 2	Minimum number to detect 3
**High quality alignments**		
Breakdancer	138	NA
GASV	72	NA
GASVPro-HQ	61	NA
Hydra-HQ	43	NA
		
**All alignments**		
GASVPro	141	286
GASVPro-Min2	141	286
Hydra	551	752

We report the minimum number of predictions required to predict two or three known inversions from a set of ten previously validated inversions [16]. NA, not applicable.

## Discussion

We introduce GASVPro, a method for SV detection that: (1) integrates both the RD signal (including the more localized beRD) and PR signal of structural variation into a single probabilistic model; (2) analyzes multiple possible read alignments using an MCMC procedure; and (3) explicitly defines uncertainty in the breakends of a variant. GASVPro is the first method to utilize a probabilistic formulation to identify generic SVs and not only copy number variants. We demonstrated that, compared to the previously published methods Breakdancer, Hydra and GASV, GASVPro has significantly higher specificity at equal or greater sensitivity in detecting known variants. Finally, our method is easily generalized to include additional signals predictive of variants.

The increased specificity and sensitivity of GASVPro demonstrates the benefit of integrating multiple signals of structural variation into a probabilistic model. In particular, read depth provides a strong signal to detect deletions and classify them as homozygous or heterozygous. As previously noted, GASVPro-HQ successfully classifies 104 of 119 deletions with known ploidy on NA12878. In contrast, methods that consider only discordant fragments, including Breakdancer, GASV and Hydra, yield more false positive predictions than GASVPro. In addition, we show that beRD is useful in increasing specificity for predicting copy-neutral inversions. Finally, our likelihood formulation provides more useful criteria for prioritizing predictions than the commonly used heuristic of the number of supporting fragments. We anticipate that including SRs will also aid in eliminating false positive predictions. In particular, the breakend polygon and beRD signal will suggest the sequence content of SRs. Thus, it will be possible to examine the data for SRs based on their sequence without exhaustive re-alignments to the reference.

The results of GASVPro demonstrate improved sensitivity when including reads with multiple possible alignments to the reference genome. However, this gain in sensitivity comes at a cost of reduced specificity as GASVPro makes many more predictions. On its surface, this is not too surprising as the inclusion of the additional lower quality alignments greatly increases the space of possible variants. The MCMC algorithm used in GASVPro is able to overcome the added ambiguity in part, with increased specificity over naïve inclusion of ambiguous alignments, but there remains a trade-off in improved sensitivity versus reduced specificity. An important caveat of this conclusion is that it is not possible to compute the actual specificity for the two sequenced human genomes, as the set of experimentally validated SVs is likely not to be the complete list of SVs in these genomes. In particular, the SVs with breakpoints in repetitive regions - those where we expect GASVPro to have some advantage - are also the hardest to predict and experimentally validate, and are thus likely greatly underrepresented in the list of experimentally validated predictions. As the lists of validated SVs become more complete, it will be possible to perform more complete benchmarking of the sensitivity and specificity of prediction methods.

The increased specificity attained by GASVPro demonstrates the benefit of including concordant coverage. An important consideration when using concordant mappings is that distinct regions of the genome will have reduced coverage for reasons unrelated to structural variation. As discussed in the Materials and methods section, repetitive sequences in the reference genome will reduce the ability of alignment software to align concordant fragments. In addition, as previously noted, there is a bias in Illumina sequencing related to the GC content of a region [14]. For the probabilistic model for deletions, we found that scaling concordant coverage according to the local mapability from the Rosetta Uniqueness Track improved sensitivity for detection. However, the use of a specific track is not essential for our model; indeed, the GASVPro code is modular and allows the user to substitute alternative models for concordant coverage and scaling. Finally, it has been previously suggested that RD is better modeled by distributions other than Poisson [50] and these could be used in place of the Poisson distribution in Equations 1 to 9.

The probabilistic method of GASVPro is formulated for a 'generic breakend' and is thus applicable to any SV class since we expect a drop in the coverage by concordant fragments at the breakends of the SV. Although deletion SVs have a stronger signal of decreased coverage throughout the region, by carefully considering the uncertainty in the location of mated breakends we identify the subtle signal of highly local drops in concordant coverage consistent with copy neutral variants such as inversions and reciprocal translocations. In this formulation, we assume 'clean' breaks in the genome, meaning there is no gain or loss of additional bases at the rearrangement junction. In practice, however, ambiguity in breakend location is likely to cause difficulties in estimating the true location and likelihood of a variant. For example, on the simulated Venter genome, coverage around the true variant breakends was significantly reduced by short indels.

As presented, our probabilistic model considered only concordant and discordant mappings; however, the model is easily generalized to include additional information about the alignments of PRs. As stated above, the SR signal can be included as part of the expected coverage around a breakend. The distribution of fragment lengths can be included when computing the likelihood of mated breakends (*a*,*b*) as each choice imposes a length on the supporting discordant fragments. Similarly, the mapping quality (or alignment score) of each mapped fragment can be incorporated into the probability function by considering the probability a chosen mapping is the correct one. We experimented with including quality scores on our simulated Illumina data set, but found this had a marginal effect on the results. However, with the addition of third-generation sequencing technologies with different error models [51], quality scores may be important.

Finally, because our probabilistic model is based on the generative processes of sequencing genomes, our model can be adapted to more general settings, such as detecting structural variation in cancer genomes. However, the extension to cancer genomes is non-trivial. In particular, to accurately analyze cancer genomes one would need to consider sample heterogeneity as the sequenced genomes are inevitably a mixture of normal and cancer genomes and possibly tumor subpopulations. In addition, our probabilistic model would need to incorporate aneuploidy by allowing more than two copies of the genomic region.

## Conclusions

Structural variation - including duplications, deletions, insertions, inversions and translocations - is an important component of genetic variation in both human and cancer genomes. Current methods for SV detection typically consider only one of several signals from resequencing data when predicting structural variation. We introduced GASVPro, a probabilistic model for identification of structural variation integrating both RD and PR signals of SVs. Compared to existing methods, GASVPro has high sensitivity in predicting known variants while reducing the number of false positives by up to 90% for deletions and 50% for inversions.

## Materials and methods

### Defining breakpoint regions with GASV

GASVPro clusters discordant PRs using the previously published program GASV [33]. The GASV algorithm explicitly represents uncertainty in the location of the endpoints of the SV, the mated breakends, by a polygon and clusters discordantly mapped fragments by utilizing a computational geometric approach for intersecting polygons. We briefly overview the approach used in GASV; for a more detailed discussion of the GASV algorithm, refer to [33].

A discordant mapping indicates a SV in the test genome defined by a novel adjacency (*a*,*b*), where positions *a *and *b *are adjacent in the test genome, but not in the reference genome (Figure 1). A single fragment alone does not uniquely specify the pair of breakends (*a*,*b*) defining the rearrangement, but rather defines uncertainty in the location of the breakends. Formally, if we assume that a discordant fragment corresponds to exactly one SV, then the mapped locations, *x *and *y*, of the fragment endpoints (without loss of generality we restrict *x *<*y*), and the breakends *a *and *b *satisfy:

$L_{\text{min}}\leq sign\left(x\right)\left(a-x\right)+sign\left(y\right)\left(b-y\right)\leq L_{\text{max}}$, (11)

where *sign*(*x*) and *sign*(*y*) are 1 if the reads align to the positive strand and have convergent orientation and -1 otherwise. Here we assume convergent orientation is when reads have opposite orientation with the left read forward and the right read reversed as in the case for Illumina sequencing technology. The inequality (Equation 11) defines a trapezoid in the plane; discordant fragments corresponding to the same SV will have overlapping trapezoids and their intersection can be used to further refine the uncertainty in breakend location as in Figure 1b.

### Concordant coverage and mapability

We consider concordant mappings when computing the likelihood of a variant because statistically significant changes in coverage indicate the presence of rearrangements relative to the reference genome. However, in addition to SVs, several local factors will affect coverage by concordant fragments.

Reads originating from duplications present in both the test and reference genome cannot be mapped to a unique position. Thus, such regions will have low coverage due to restrictions in local mapability. To adjust for variable mapability throughout, in the deletion model we scaled the number of concordantly mapped fragments using The Rosetta Uniqueness Track. The Rosetta Uniqueness Track, created by John Castle at Rosetta Inpharmatics (Merck; UCSC Genome Browser), quantifies mapability by considering a 35-bp tiling of the genome and determining which 35-mers will have a unique mapping to the reference genome with the Burrows-Wheeler aligner (BWA) mapping tool.

For an interval , let R(I) be the fraction of uniquely mapable bases in according to the Rosetta Uniqueness Track and n(I) be the number of observed concordant fragments whose mappings overlap . In our analysis we consider the scaled concordant coverage $\hat{n}\left(I\right)$, where:

(12) $$\hat{n}\left(I\right)=\frac{n\left(I\right)}{\alpha +\beta R\left(I\right)},$$

where we use α=0.3 and β=0.7. Notice, when the interval  does not have compromised mapability, that is, R(I)=1, we do not adjust the number of observed fragments, $\hat{n}\left(I\right)=n\left(I\right)$.

Note that in our analysis we do not scale the number of discordant fragments. In practice we found an abundance of discordant fragments mapping to regions of very low-mapability and scaling the number of discordant fragments led to an abundance of false positive predictions. Finally, we utilized a heuristic when computing the likelihood of SVs. If the concordant coverage for a breakpoint or interval was in the top 0.01% according to the Poisson model, we automatically assigned C(B)=0. Since under the Poisson model extremely high coverage by concordant fragments occurs with low probability, this threshold further restricts the region considered to represent SV endpoints. In such cases, we expect coverage by concordant fragments to be decreased compared to the rest of the genome.

### Prediction uncertainty and the double uncertainty metric

Most studies determine if predictions match a known variant by overlapping a predicted genomic interval with the interval reported for the known variant. However, the criteria for 'overlap' differs among methods. For example, Chen *et al*. [32] considered a match when the intersection of the intervals is at least 50% of the union of the two or if the predicted interval entirely contains the known variant. While Hormozdiari F *et al*. [42] reported a deletion as matching a known variant if they had 50% reciprocal overlap and considered any overlap between an inversion and known variant. Although these criteria do eliminate some types of spurious identification, the inherent weakness of these metrics is that they do not unambiguously represent the underlying uncertainty in the predicted or reported variants.

We introduce a criteria for overlap, the 'double uncertainty' metric, that explicitly represents uncertainty in both the coordinates of known variants, reference uncertainty, and predictions, prediction uncertainty. We say a prediction and known variant overlap if the pairs of intervals specifying uncertainty in their coordinates do. Formally, ∈≥0 specifies the prediction uncertainty and δ≥0 is the reference uncertainty. That is, for the predicted SV, the left breakend is predicted to lie in the interval [x-∈,x+∈] and the right breakend in the interval [y-∈,y+∈]; similarly, the reported known variant has left breakend in the interval, [a-δ,a+δ] and right breakend in the interval [b-δ,b+δ]. A predicted SV overlaps a known variant in the double uncertainty metric if both of the following are satisfied:

1. [x-∈,x+∈]∩[a-δ,a+δ]≠∅

2. [y-∈,y+∈]∩[b-δ,b+δ]≠∅

We provide illustrations of the double uncertainty metric in Additional file 1. We illustrate the conversion from output formats from different SV programs to use in the comparison in Figure A4 and overlap in the double uncertainty metric in Figure A5 in Additional file 1.

In practice, the reference uncertainty, , and prediction uncertainty, , reflect limitations on the technology used, such as fragment size, but may also include ambiguity inherent in a breakend within a repetitive region. We use prediction uncertainty $\in =L_{\text{max}}/2$ to reflect the sequencing process. We base the reference uncertainty on the specific data set and technology used to obtain the known variants. For the Venter simulated deletions, reference uncertainty is 0 because these variants are specified to the breakpoint. For variants from fosmid mappings of NA12878 or NA18507 we use fosmid mappings reported by Kidd *et al*. [16] to determine the breakend polygon with GASV, and use the uncertainty directly from these polygons.

### Markov chain Monte Carlo procedure

Given an alignment matrix *A*, we define a Markov chain  over the space of mapping matrices  that has P(M|A) as its stationary distribution. We use the Metropolis Hastings algorithm to define transition probabilities between matrices  and $M^{\prime }$, $p\left(M,M^{\prime }\right)$. The probability of transitioning between states depends on two terms: proposing a move with proposal distribution $q\left(M,M^{\prime }\right)$ and accepting this move with probability $\alpha \left(M,M^{\prime }\right)$. That is:

$$p\left(M,M^{\prime }\right)=q\left(M,M^{\prime }\right)\alpha \left(M,M^{\prime }\right).$$

If the proposal distribution $q\left(M,M^{\prime }\right)$ yields an irreducible and aperiodic Markov chain, then using the acceptance probability of the Metropolis Hastings procedure:

(13) $$\alpha \left(M,M^{\prime }\right)=\text{min}\left\{1,\frac{q\left(M^{\prime },M\right)P\left(M^{\prime }|A\right)}{q\left(M,M^{\prime }\right)P\left(M|A\right)}\right\}$$

results in convergence of  to the stationary distribution P(M|A). The first step in our MCMC procedure (Figure A2 in Additional file 1) is to stay at the same mapping matrix  with probability 1/2. This self-edge guarantees aperiodicity, but irreducibility depends on the set  of possible moves. We developed several classes of moves (Figure A3 in Additional file 1) to explore the space of mapping matrices that yield irreducibility. The first move consists of naively moving a fragment from one mapping to another:

Naive (N):

Select a row *i *with uniform probability:

If there is a *j *such that $m_{ij}=1$ set $m_{ij}=0$. If there exists *k *≠ *j *such that $a_{ik}=1$, then with probability $\left(1-p_{err}\right)$ select a *k *uniformly and set $m_{ij}=1$. Otherwise, leave $m_{ik}=0$ for all *k*.

If $m_{ij}=0$ for all *j*, with uniform probability select a *j *where $a_{ij}=1$ and set $m_{ij}=1$.

Notice that the Naive (N) move always changes the mapping matrix. If  consists of only class N moves, then the chain M(Γ) satisfies irreducibility because any two mapping matrices  and $M^{\prime }$ may be reached from one another by a series of class N moves. Thus, the Markov chain M(Γ) with  equal to the all class N moves will yield the stationary distribution P(M|A). However, we found empirically that the mixing time of a Markov chain with only a class N move was long (Section A2 in Additional file 1). Thus, we define three additional moves, which empirically yielded improved mixing times. (Recall from the main text that, for an assignment matrix  and associated mapping matrix , V(M) is the number of SVs with positive support, $R_{j}\left(A\right)$ is the set of rows in  with a 1 in the column *j *and $S_{j}\left(M\right)=\left|R_{j}\left(M\right)\right|$ is the total support for a variant *j*.)

Remove a single column (Z): this move zeroes out a column of *M*:

With probability $\frac{1}{V\left(M\right)}$, select a non-zero column *j*.

For all $i\in R_{j}\left(A\right)$:

If $m_{ij}=1$, set $m_{ij}=0$. If there exists *k *≠ *j *such that $a_{ik}=1$, with probability $\left(1-p_{err}\right)$ uniformly select a column *k *and set $m_{ik}=1$. Otherwise, leave $m_{ik}=0$ for all *k*.

Revive a zero column ($\bar{Z}$): This move adds support to a zero column of *A*:

With probability $\frac{1}{\left(n-V\left(M\right)\right)}$, where *n *is the total number of variants in *V*, select a zero column *j*, that is, a column *j *with $S_{j}\left(M\right)=0$.

While $m_{ij}=0$ for all $i\in R_{j}\left(A\right)$:

For each *i*, with probability 1/2, set $m_{ij}=1$ and $m_{ik}=0$ for all *k *≠ *j*.

Swap columns (S): this move swaps some entries of two columns of *A*:

Let $R_{jk}\left(A\right)=R_{j}\left(A\right)\cap R_{k}\left(A\right)$. With probability:

$$\frac{\left|R_{ij}\left(A\right)\right|}{\underset{j^{\prime }}{\sum }\underset{k^{\prime }:k^{\prime }>j^{\prime }}{\sum }\left|R_{j^{\prime }k^{\prime }}\left(A\right)\right|},$$

select a pair of columns (j,k) conditional on at least one column having non-zero entries.

For all $i\in R_{ij}\left(A\right)$:

If $m_{ij}=1$ or $m_{ik}=1$, then with probability 1/2, swap $m_{ij}$ and $m_{ik}$.

Repeat if necessary to ensure at least one entry is swapped between columns *j *and *k*.

The last step in formalizing the Markov chain is to compute the acceptance probabilities. As described above, the acceptance probability depends on the proposal distribution and the probability of the mapping matrix. In fact, since the acceptance probability depends on only the ratio $\frac{P\left(M^{\prime }|A\right)}{P\left(M|A\right)}$, the computation is simplified to considering only the columns (variants) and rows (fragments) that differ between the matrices. This ratio is simple for class N and S moves since a Naive move alters exactly one row and at most two columns and a Swap move alters exactly two columns. Although in the worst case Z and $\bar{Z}$ may alter every row and column of the mapping matrix, this is quite rare in practice.

In order to compute the proposal distribution, we need to consider all ways to transition between mapping matrices $M^{\prime }$ and . First, note that all move classes result in a new matrix $M^{\prime }$. Thus, the probability of a self-loop is always fixed at 1/2. Second, note that in many cases different move types will create the same resulting mapping matrix. For example, a class Z move on a variant with only a single supporting fragment is the same as a class N move on that supporting fragment. Thus, the proposal distribution $q\left(M^{\prime },M\right)$ and $q\left(M,M^{\prime }\right)$ must consider all possible move types. We use $q_{N},q_{S},q_{Z}$ and $q_{\bar{Z}}$ to distinguish between the proposal distribution conditional on a move class.

A class N move alters the assignment of exactly one row. Let  be the number of rows (that is, discordant fragments), then probability of proposing $M^{\prime }$ with a class N move will be one of the following values:

1. If the altered row had only one possible non-zero entry in *A*, that is $\left|R_{i}\left(A\right)\right|=1$, $q_{N}\left(M,M^{\prime }\right)=\left(1/F\right)$,

2. If $m_{ij}=1$ and $m_{ik}^{\prime }=1$ for $m_{ij}\in M$ and $m_{ik}^{\prime }\in M^{\prime }$, then $q_{N}\left(M,M^{\prime }\right)=\left(1/F\right)\frac{\left(1-p_{err}\right)}{\left|R_{i}\left(A\right)\right|-1}$,

3. If $m_{ij}=1$ and $m_{ik}^{\prime }=0$ for $m_{ij}\in M$ and $m_{ik}^{\prime }\in M^{\prime }$ for all *k*, then $q_{N}\left(M,M^{\prime }\right)=\left(1/F\right)p_{err}$,

4. If $m_{ij}=0$ and $m_{ik}^{\prime }=1$ for $m_{ij}\in M$ for all *i *and $m_{ik}^{\prime }\in M^{\prime }$, then $q_{N}\left(M,M^{\prime }\right)=\frac{\left(1/F\right)}{\left|R_{i}\left(A\right)\right|},$

and 0 if no class N move is possible.

A class Z move results in a single empty variant. Let V(M) be the number of non-empty columns. The proposal distribution of a class *Z *move depends on selecting the column, with probability 1/V(M) and reassigning the rows to either errors, with probability $p_{err}$, or another mapping, with probability $\frac{\left(1-p_{err}\right)}{\left|R_{i}\left(A\right)\right|-1}$. Let *x *be the number of rows moved to an error, when another mapping is possible, and *y *be the set of rows moved to another mapping given that at least two mappings are possible, then:

(14) $$q_{Z}\left(M,M^{\prime }\right)=\frac{1}{V\left(M\right)}p_{err}^{x}\overset{\left|y\right|}{\underset{i=1}{\prod }}\left(\frac{\left(1-p_{err}\right)}{|R_{y\left(i\right)}\left(A\right)|-1}\right)$$

and 0 if no class Z move is possible.

In a class $\bar{Z}$ move, all altered rows are moved to the same originally empty column. A class $\bar{Z}$ move depends on selecting an empty column to add to, with probability 1/(n-V(M)), and moving entries from other columns. Let *j *be the column that was selected to be added to, then $\left|R_{j}\left(A\right)\right|$ is the total number of rows that could be assigned to *j*. We first select the number of entries *k *to move to column *j*, conditional on at least one entry changing. Then, the proposal distribution is given by:

(15) $$q_{\bar{\text{Z}}}\left(M,M^{\prime }\right)=\frac{\text{Prob(Moving }k\text{ entries)}}{\left(\begin{array}{c} \left|R_{j}\left(A\right)\right|\\ k \end{array}\right)\left(n-V\left(M\right)\right)},$$

and 0 if no class $\bar{Z}$ move is possible.

The proposal distribution for a class S move is nearly identical to the class $\bar{Z}$ move, except we need only consider the probability of picking the two columns instead of picking a single non-empty column. As before we define $q_{S}\left(M,M^{\prime }\right)=0$if no class *S *move is possible.

Finally, for the full proposal distribution:

(16) $$q\left(M,M^{\prime }\right)=\chi \left(M,M^{\prime }\right)\left(q_{N}\left(M,M^{\prime }\right)+q_{Z}\left(M,M^{\prime }\right)+q_{\bar{\text{Z}}}\left(M,M^{\prime }\right)+q_{S}\left(M,M^{\prime }\right)\right),$$

where $\chi \left(M,M^{\prime }\right)$ is an appropriate weighting factor based on which moves are possible. For example, if the transition from  to $M^{\prime }$ is possible with all move types, then $\chi \left(M,M^{\prime }\right)=1/4$.

We now formally demonstrate our Markov chain converged to P(M|A) given in Equation 10. As described above, our chain is aperiodic and irreducible, since there is a nonzero probability of moving from any one state to any other state in a finite number of steps. A finite state, irreducible and aperiodic Markov chain has a unique stationary distribution  and this distribution satisfies the detailed balance condition:

(17) $$\pi \left(M\right)p\left(M,M^{\prime }\right)=\pi \left(M^{\prime }\right)p\left(M^{\prime },M\right)$$

Here, transition probability $p\left(M,M^{\prime }\right)$ depends on two terms, proposing a move from state  to $M^{\prime }$ with proposal distribution $q\left(M,M^{\prime }\right)$ and accepting this move with probability $\alpha \left(M,M^{\prime }\right)$: $p\left(M,M^{\prime }\right)=q\left(M,M^{\prime }\right)\alpha \left(M,M^{\prime }\right)$. We show that the acceptance probability satisfies the detailed balance condition in Equation 17. Without loss of generality assume $\alpha \left(\theta ,\theta^{\prime }\right)=\frac{q\left(\theta^{\prime },\theta \right)\pi \left(\theta^{\prime }\right)}{q\left(\theta ,\theta^{\prime }\right)\pi \left(\theta \right)}$, then $\alpha \left(\theta^{\prime },\theta \right)=1$. Thus:

$$\begin{array}{llll} \pi \left(\theta \right)p\left(\theta ,\theta^{\prime }\right) & =\pi \left(\theta \right)q\left(\theta ,\theta^{\prime }\right)\alpha \left(\theta ,\theta^{\prime }\right)\; & & \;\\ & =\pi \left(\theta \right)q\left(\theta ,\theta^{\prime }\right)\frac{q\left(\theta^{\prime },\theta \right)\pi \left(\theta^{\prime }\right)}{q\left(\theta ,\theta^{\prime }\right)\pi \left(\theta \right)}\; & & \;\\ & =q\left(\theta^{\prime },\theta \right)\pi \left(\theta^{\prime }\right)\; & & \;\\ & =\pi \left(\theta^{\prime }\right)\underset{p\left(\theta^{\prime },\theta \right)}{\underset{︸}{q\left(\theta^{\prime },\theta \right)\overset{1}{\overset{︷}{\alpha \left(\theta^{\prime },\theta \right)}}}}\; & & \;\\ & =\pi \left(\theta^{\prime }\right)p\left(\theta^{\prime },\theta \right)\; & & \;\\ & \; & \end{array}$$

### Efficient sampling of mapping matrices

A practical difficulty in sampling from the space of mapping matrices is the high dimension of the sampling space with millions of discordant fragments and hundreds of thousands of potential variants in the genomes in this study. However, we are still able to efficiently explore the space of mapping matrices by subdividing potential variants and discordant fragments into independent subsets and sampling instead over sub-matrices of  (Figure 3).

Let  be a bi-partite graph defined by disjoint sets of vertices corresponding to the fragments, , and variants . There is an edge from a vertex f∈F to V∈Vif there is a mapping of *f *that supports the SV . A connected component *c *of *G *corresponds to a sub-matrix  of *A *and  of *M*. The moves employed in our MCMC procedure only modify assignments belonging to a single connected component of *G*. Further, since:

(18) $$\underset{c}{\prod }P\left(M_{c}|A_{c}\right)=P\left(M|A\right),$$

we subdivide our sampling by separately considering each connected component in *G *(Figure 3). Sampling separately over mapping sub-matrices $M_{c}$ for each *c *and combining results is equivalent to sampling over the full space of mapping matrices because each move in the former has an equivalent move with equal probability in the latter and vice versa. Further, because we never compute *P*(*M*|*A*) alone, but only the ratio *P*(*M*'|*A*)/*P*(*M*|*A*) for a proposed mapping matrix *M*', Equation 18 is more general than needed. Thus, we instead verify the following sufficient condition:

(19) $$\frac{\underset{c}{\prod }P\left({M^{\prime }}_{c}|A_{c}\right)}{\underset{c}{\prod }P\left(M_{c}|A_{c}\right)}=\frac{P\left(M^{\prime }|A\right)}{P\left(M|A\right)}$$

As stated above, each move only affects one connected component. Let $c^{\prime }$ be the component affected by the move, then the ratio on the left hand side of Equation 19 becomes $P\left({M^{\prime }}_{c^{\prime }}|A_{c^{\prime }}\right)/P\left(M_{c^{\prime }}|A_{c^{\prime }}\right)$, since *c*' is the only component changed. Thus, we replace the left hand side in Equation **19:**

(20) $$\frac{P\left({M^{\prime }}_{c^{\prime }}|A_{c^{\prime }}\right)}{P\left(M_{c^{\prime }}|A_{c^{\prime }}\right)}=\frac{P\left(M^{\prime }|A\right)}{P\left(M|A\right)}$$

To see that Equation 20 holds as equality, recall that *P*(*M*'|*A*)/*P*(*M*|*A*) is computed over rows and columns of *M *and *M*' except the term $\eta e^{-\eta V\left(M\right)}$, which only depends on the total number of variants with positive support. Again, let *c*' be the component affected by the move. Thus, when computing $P\left(M^{\prime }|A\right)/P\left(M|A\right)$, every term corresponding to rows or columns that belong to components other than *c*' are cancelled. Moreover the ratio $\frac{\eta e^{-\eta V\left(M^{\prime }\right)}}{\eta e^{-\eta V\left(M\right)}}=e^{-\eta \left(V\left(M\right)-V\left(M^{\prime }\right)\right)}$ depends on only the number of columns whose support changes. Thus, this ratio also depends on only the columns affected by the move. Therefore, $P\left(M^{\prime }|A\right)/P\left(M|A\right)$ also becomes $P\left(M_{c^{\prime }}^{\prime }|A_{c^{\prime }}\right)/P\left(M_{c^{\prime }}|A_{c^{\prime }}\right)$ and Equation 20 is satisfied.

### Defining the predicted variants

As indicated in the main text, we considered several different procedures for reporting a final set of predictions from the mapping matrices *M *sampled during the MCMC procedure. The simplest method is to consider a single mapping matrix that maximizes *P*(*M*|*A*) as the truth and report the resulting variants. However, we found a useful procedure was to consider the entire set of mapping matrices sampled during the Markov chain .

We first consider a variant-based method for analyzing the Markov chain . We note that the likelihood ratio, Λ, was a useful test statistic to prioritize variants when the set of mappings was fixed. We generalize Λ(V,M) to be the likelihood ratio of a variant according to a specified mapping matrix and seek the likelihood of a variant over the entire space of mapping matrices:

(21) Λ(V)= ∑Λ(V,M)P(M|A)

Assuming the Markov chain  has converged, we can approximate Λ(*V*) from the chain:

(22) $$\Lambda \left(V\right)\approx \frac{1}{N}\overset{N}{\underset{i=1}{\sum }}\Lambda \left(V,M_{i}\right)$$

We also analyzed a fragment-based approach by considering each fragment independently over each mapping matrix *M *sampled in the Markov chain . For each fragment *i *and mapping *j *we define the average support for this mapping as:

(23) $${\bar{m}}_{ij}=P\left(m_{i,j}=1|A\right).$$

(Note that ${\bar{m}}_{ij}$ is directly determined during the MCMC sampling procedure.) If a fragment has the same assigned mapping for a majority of the Markov chain (that is, ${\bar{m}}_{ij}\geq 0.5$), we are inclined to label that alignment the 'true mapping'. That is, we define a matrix ${\bar{M}}^{\left(\tau \right)}=\left[{{\bar{m}}_{ij}}^{\tau }\right]$ where:

$${{\bar{m}}_{ij}}^{\tau_{}^{}}\left\{\begin{array}{c} 1,\;\text{if }{\bar{m}}_{ij}\geq \tau ,\text{ for some }\tau >0.5\;\\ 0,\;\text{otherwise } \end{array}\right)$$

Notice that ${\bar{M}}^{\left(\tau \right)}$ has two favorable properties: we consider at most one mapping for each fragment, and we exclude fragments that do not strongly support a single SV. In the results we present we define our final set of predictions by ${\bar{M}}^{\left(\tau \right)}=\left[{{\bar{m}}_{ij}}^{\tau }\right]$ and report variants based on their likelihood ratio Λ according to this mapping matrix. However, over all datasets studied, we found only minor differences in the ROC curves for three different sets of predictions (see Figure A4 in Additional file 1 for a comparison).

### Mapping reads

We analyzed alignments to two human genomes (NA18507 and NA12878) and a simulated human chromosome. In all cases, we used two sets of alignment data: a high quality data set and a low quality data set. The high quality data set consisted of fragments with a clear and unique mapping to the reference genome. For human genomes NA18507 and NA12878, the reported mappings (from [14] and [44], respectively), were taken as the high-quality set. For the simulated data for Venter chromosome 17, we mapped reads to the reference chromosome 17 with BWA [52] to determine the high quality unique mappings.

The low quality alignments were obtained by using NovaAlign [48] to realign reads not belonging to a uniquely mapped pair. We allowed up to 100 alignments per read, but to eliminate fragments from highly repetitive data, we removed all fragments with more than 100 alignments genome-wide. Although our low quality alignments contained ambiguous fragments, many were low quality unique mappings. For NA18507, 516,941 out of 888,868 fragments included in the low quality set had unique mappings. For NA12878, 69,388 out of 157,842 fragments had unique mappings. For both sets of alignments, we removed concordant fragments, and retained all alignments with mapped distance ≤500 kb and with mapping quality >10.

For Breakdancer and GASV, results were only given on the high quality mappings. For GASVPro and Hydra, the suffix '-HQ' specifies results on the high quality datasets; results without the suffix were on the combined high and low quality datasets.

#### Running GASVPro

##### Runtime analysis

We now discuss details of the GASVPro algorithm; after identifying the set of discordant and concordant mappings, there are three steps in the pipeline of GASVPro: (1) clustering discordant mappings with GASV (*O*(*n*log*n*) in the number of discordant fragments); (2) determining concordant coverage over each breakend polygon, (*O*(*C*log*C*) where *C *the number of concordant fragments); and (3) running the MCMC sampling procedure.

When running the MCMC procedure on each connected component (Figure 3), we utilize a fixed number of burn-in iterations (10^5^) and sampling steps (9 × 10^5^) based on heuristics developed in analyzing the simulated data. The complexity of the MCMC depends on selecting a move and deciding whether to accept the proposed move, each of which depends on the size of the connected component considered. With *m *discordant fragments and *n *variants in a connected component, the time to select a move, over all possible move types, is $O\left(m+n+n^{2}\right)$. Determining if a move is accepted depends on the number of altered variants and fragments; in the worst case all variants and fragments could be altered *O*(*n*+*m*), but in practice the total number of modified cases is quite small.

##### MCMC parameters and considerations

The parameter λ varied with the coverage of the data; we used λ = 0.3 for the simulated Venter chromosome and NA12878, and λ = 0.6 for NA18507. Our final results were not sensitive to the exponential prior on the number of variants. However, this term may be useful in other analyses. As discussed in Additional file 1, when *η *is large, *P*(*M*|*A*) is dominated by the exponential distribution $\eta e^{-\eta V\left(M\right)}$, which is maximized when the number of variants is minimized. In addition, for the genomes we studied we further restricted the space of mapping matrices by fixing the mapping for fragments with a unique assignment in the genome. As such, the MCMC procedure would transition between possible mappings for only truly fragments with multiple possible alignments. Such a heuristic greatly reduces the computation time of the MCMC by significantly reducing the total space to sample.

Finally, additional considerations were made in the analysis of NA18507. A combination of high coverage and short reads (37 bp) resulted in nearly half a million predicted deletions and several extremely large connected components ≥2 × 10^5 ^clusters with over 10^6 ^fragments. Because of computational difficulties in analyzing these clusters, along with difficulties in determining convergence of the MCMC procedure, we eliminated all mappings to the centromeres and retained only mappings that indicated a deletion larger than 1,000 bp. (In the results discussed in the main text, all methods were compared on this same reduced set of fragments.) After these measures, there remained six connected components where the number of edges in the graph exceeded 10^6^. In analyzing these connected components, we simply assigned fragments with unique mappings.

#### Pruning predicted structural variants

Results from all methods were pruned in a post-processing step to eliminate redundant predictions. First, predictions from all methods were converted to intervals. For GASV, a breakend polygon *B *was converted into an interval $I\left(B\right)=\left[a_{\text{max}},b_{\text{min}}\right]$, where $a_{max}=\underset{a}{\text{arg}\text{max}}\left\{\left(a,b\right)\in B\right\}$ and $b_{\text{min}}=\underset{b}{\text{arg}\text{min}}\left\{\left(a,b\right)\in B\right\}$. For Breakdancer we considered the reported interval [*x*,*y*] and for Hydra we considered the interval [*IE*,*OS*] (Figure A5 in Additional file 1).

Two predictions were said to be redundant if the intersection of their intervals was at least 50% of the union or if one interval contained the other. In such cases, for GASV, Breakdancer and Hydra, the prediction with more supporting fragments was retained. For GASVPro, the prediction with greater likelihood according to the respective model was retained.

#### Known variants

As discussed in the text, we compared predictions to sets of known SVs with the double uncertainty metric. Importantly, this metric considers uncertainty in the location of both the prediction and known variant.

For the simulated Venter genome, we compare predictions to the set of deletions and inversions detailed in [47]; there were 4 inversions and we used the 124 deletions with length ≥125 bp. When comparing predictions to known variants, we use reference uncertainty δ = 0 because the true location of the breakpoints is known exactly.

For both genomes, NA12878 and NA18507, we compared predictions from each method to two sets of validated variants. The first was from a fosmid sequencing study [16] and the second from the 1000 Genomes Project pilot study [44]. Although the combined set of variants likely contained duplicates, to maximize sensitivity we did not attempt to reduce these sets by eliminating predictions reported by both studies.

A fosmid sequencing study validated hundreds of inversions and deletions [16]. We considered only the subset of predictions that were validated in the same individuals (NA18507, NA12878). As previously reported, several of the predictions from the original study did not have a common breakpoint region defined by fosmid mappings [33]. Thus, we restricted the validated set to the 93 deletions and 10 inversions for NA18507 and 151 deletions and 23 inversions for NA12878 that corresponded to clusters of at least two fosmids. In comparisons, we utilized the inherent uncertainty in breakend polygons [33] as the reference uncertainty.

The 1000 Genomes Project pilot study reported validated deletion variants as well as the individuals to whom they belonged [44]. (Note that the pilot study did not report inversion SVs.) We separated validated variants that were identified by PR mapping and restricted to deletions that were larger than 5 kb. The final set represented a total of 118 deletions for NA18507 and 139 deletions for NA12878. Because many next-generation sequencing libraries were used in predicting these variants, we used δ = 200 as an approximation for the prediction uncertainty in these variants.

## Abbreviations

beRD: breakend read depth; bp: base pair; BWA: Burrows-Wheeler aligner; GASV: Geometric Analysis of Structural Variation; MCMC: Markov chain Monte Carlo; PR: paired read; RD: read depth; ROC: receiver operating characteristic; SR: split read; SV structural variant.

## Competing interests

The authors declare that they have no competing interests.

## Authors' contributions

SS, SO and BJR conceived and designed the method. SS, SO, LP and HW developed code and generated datasets. SS, BJR, LP and SO performed the analyses. SS and BJR wrote the manuscript. All authors read and approved the final manuscript.

## Supplementary Material

Additional file 1 **An Appendix containing additional figures, discussion of MCMC properties and comparison of clustering methods**. Click here for file
