# Supplementary material for: An integrative probabilistic model for identification of structural variation in sequencing data
Source: Genome Biol. 2012 Mar 27;13(3):R22. doi: 10.1186/gb-2012-13-3-r22 (PMC3439973; doi:10.1186/gb-2012-13-3-r22)
Supplement: Additional file 1 — An Appendix containing additional figures, discussion of MCMC properties and comparison of clustering methods. [file gb-2012-13-3-r22-S1.PDF]

Appendix:
An Integrative Probabilistic Model for Identification of Structural Variation in Sequencing Data

Suzanne S. Sindi*[1,2], Selim Onal[3] , Luke C. Peng[3] , Hsin-Ta Wu[3] , Benjamin J. Raphael*[1,3]

[1]Center for Computational Molecular Biology
[2]Department of Molecular Biology, Cellular Biology and Biochemistry
[3]Department of Computer Science
Brown University, Providence, RI 02912 USA.

Email: Suzanne S. Sindi*- Suzanne_Sindi@Brown.edu; Benjamin J. Raphael*- braphael@cs.brown.edu;

*Corresponding author

Section A1: Additional Figures

As referenced from the main text, we provide additional figures to support the presentation of the GASVPro algorithm and the double uncertainty metric.

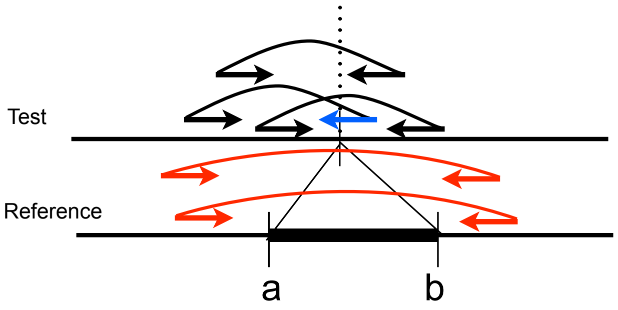

Figure A1: **Scaling Coverage of Discordant Mappings.** Coverage should be scaled for fragments containing a breakpoint. A fragment containing a breakpoint will be mapped to the reference genome only if the breakpoint is not contained in the reads. Here three fragments contain a deletion relative to the reference genome. However because the blue read contains the breakpoint itself, the read will not map uniquely with high quality to the reference genome. Because fragments whose reads contain the breakpoint will not be mapped, there is reduced coverage of discordant mappings. The expected number of fragments covering a breakpoint is $\lambda_d = \lambda\,(L_{avg} - 2 \times \text{read length})$, where L_{avg} is the average fragment length.

Input: A set Γ of possible moves

Initialization: Select a random assignment matrix M_0 by setting for all i, $m_{ij} = 1$ for exactly one j such that $a_{ij} = 1$.

Iteration: For $t = 1, 2, ...,$ obtain M_{t+1} from M_t as follows:

1. With probability 1/2, set $M_{t+1} = M_t$.
2. Otherwise, select a move γ from Γ by first selecting a move class with uniform probability and then selecting a move from this class.
3. Define a new assignment matrix $M' = \gamma(M_t)$.
4. With probability $\alpha(M_t, M')$, set $M_{t+1} = M'$. Otherwise, set $M_{t+1} = M_t$.

Figure A2: **MCMC algorithm.** Our MCMC algorithm begins by selecting a random initial mapping matrix and sampling over the space of possible mapping matrices with the set of moves Γ (see Figure A3). As shown in the Methods, our Markov Chain is aperiodic and irreducible and thus we will asymptotically converge to sampling mapping matrices M according to $P(M|A)$.

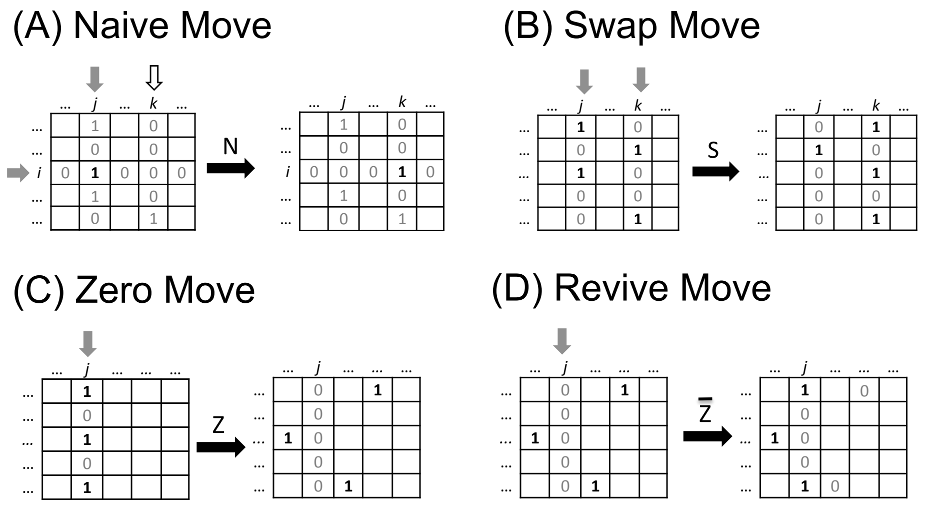

Figure A3: **MCMC Moves.** As described in Methods, we explore the space of mapping matrices with four different moves: (A) Naive (N), randomly selects a new mapping for a fragment; (B) Swap (S), swapping mappings for two potential variants that share fragments; (C) Zero (Z), removing all support for a variant; (D) Revive ($\bar{Z}$), adds some support to a currently unsupported variant.

Section A2: Convergence of Markov Chain Monte Carlo Procedure

As presented in the main text, we presented an MCMC procedure (Figure A2) to explore the space of mapping matrices proportional to a desired stationary distribution:

$$P(M|A) = \max_{\mathbf{C}} P(M, \mathbf{C}|A),$$

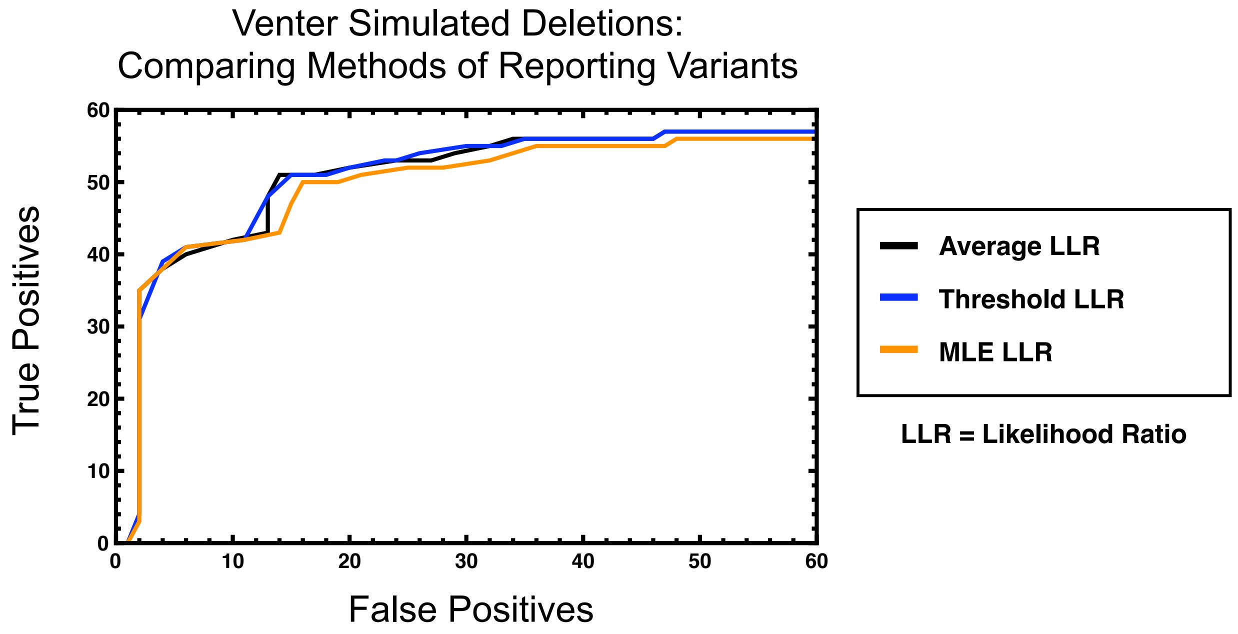

Figure A4: **Comparison of Methods to Report Variants from MCMC Procedure.** ROC curves comparing different reporting methods for GASVPro on the deletions from Venter chromosome 17. All simulations methods analyzed the same set of mappings, were post-processed in an identical fashion and compared to the known coordinates with the double uncertainty metric (see Methods).

where

$$P(M, \mathbf{C}|A) \propto \eta e^{-\eta \mathcal{V}(M)} \prod_{j:\mathcal{S}_j(M)>0} P(V_j(M)|C_j(M)) \prod_{i:\gamma_i(M)=0} p_{err}.$$

We now discuss some properties of our sampling procedure and demonstrating convergence of our sampling procedure on simulated datasets. In particular, we demonstrate that the addition of moves of class S, Z and $\bar{\text{Z}}$ (Figure A3) positively impact convergence.

First, note that the choice of values for parameters η and λ in $P(M|A)$ results in qualitatively different instances of our problem. Thus, although a sampling procedure with only class N moves is guaranteed to converge to $P(M|A)$, the time to convergence may be impacted by the choices for η and λ.

If η is large, then $P(M|A)$ is dominated by the exponential distribution $\eta e^{-\eta \mathcal{V}(M)}$, which is maximized when $\mathcal{V}(A)$, the number of variants, is minimized. Thus, the mapping matrices matrices with high probability will be those with the fewest number of non-zero columns. (Note this is similar to the classic Set Cover problem where the sets correspond to the fragments that can support a potential $\mathcal{R}_j(A)$.) In these cases, we expect that at MCMC procedure using moves from class Z and $\bar{\text{Z}}$, which always change the total number of variants, will converge faster than one using only class N or S moves. We verified this expectation with simulated studies of convergence. In Figure A6(A) we show the log-likelihood of the sampling procedure according to the iteration of the MCMC procedure; the log-likelihood increases faster when class Z and $\bar{\text{Z}}$ moves are included.

In contrast, for instances with much smaller η set-cover like solutions are no longer favorable. Thus, $P(M|A)$ will depend more strongly on the Poisson coverage of the predicted variants themselves. In these cases, we observe that Z and $\bar{\text{Z}}$ moves or even class S moves alone will sample the space efficiently (see Figure A9(B) below).

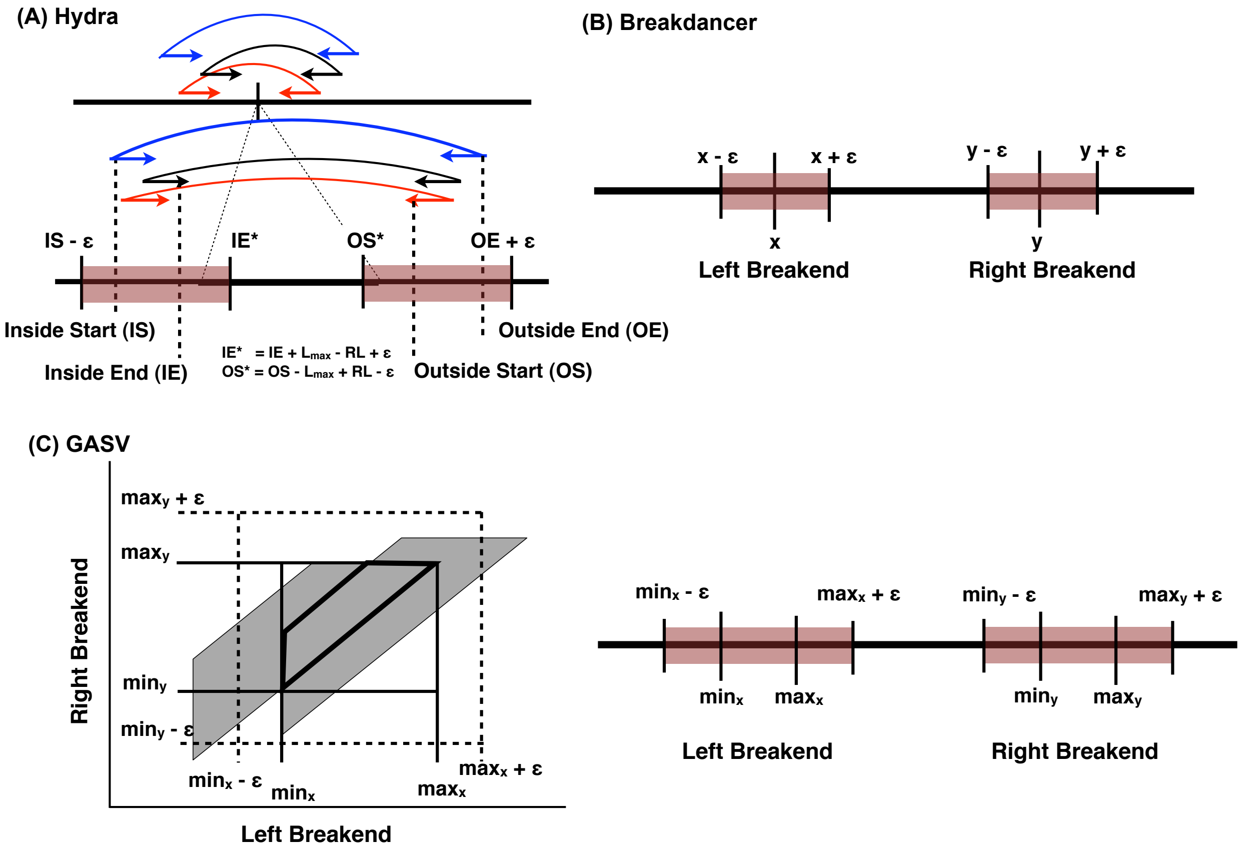

Figure A5: **Prediction Uncertainty for Each Method.** We illustrate with shaded pink the prediction uncertainty for each of the methods studied. Because each method reports structural variant predictions in a distinct manner, we define the region of uncertainty separately for each method. Hydra reports a structural variant in terms of the positions of reads in a cluster, Breakdancer reports a single coordinate and GASV reports the uncertainty in the position of the mated breakends. In each case, we allow for uncertainty in the reported prediction in accordance with the data, read length RL, fragment length distribution $L_{\max}$. The parameter ϵ is the *prediction uncertainty* and is set to $L_{\max}/2$ in our analyses. (When analyzing results for NA12878 Pilot 2, $\epsilon = \overline{L_{\max}}/2$ where $\overline{L_{\max}}$ is the average $L_{\max}$ over all sequenced libraries.)

Section A3: Comparison of Clustering Algorithms

The clustering algorithms employed by distinct methods can greatly impact the number of false positive predictions. The GASV algorithm will only cluster discordant fragments supporting the same novel adjacency. The more aggressive approach of some competing methods (see Figure A10) makes it difficult to interpret the number of discordant fragments in a prediction as the distribution of fragments will no longer follow the Poisson model of coverage. In addition, other methods may employ ad-hoc procedures for merging potential variants that result in reported coordinates that are not close to the true breakends (Figure A11).

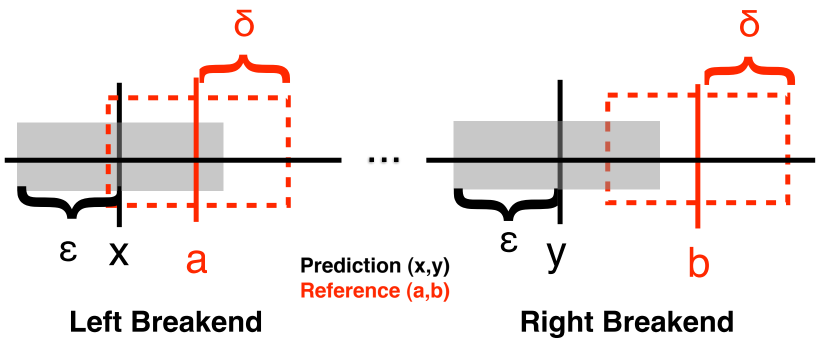

Figure A6: **Overlapping with the Double Uncertainty Metric.** We illustrate and definition of overlap between a predicted structural variant (grey boxes) and a known variant (dashed red boxes). A predicted structural variant (x, y) is said to overlap a known variant (a, b) when there is at least one possible novel adjacenty, pair of mated breakends, consistent with both estimates of uncertaintity for the left and right breakend. In this example the prediction uncertainty is $\epsilon > 0$ and thus the left and right breakends are predicted to lie within $[x - \epsilon, x + \epsilon]$ and $[y - \epsilon, y + \epsilon]$ respectively (grey boxes). Similarly, uncertaintity in the known variant is $\delta > 0$ and thus the true left and right breakends lie within $[a - \delta, a + \delta]$ and $[b - \delta, b + \delta]$ respectively. In this case (x, y) overlaps known variant (a, b) according to the double uncertainty metric because, $[x - \epsilon, x + \epsilon] \cap [a - \delta, a + \delta] \neq \emptyset$ and $[y - \epsilon, y + \epsilon] \cap [b - \delta, b + \delta] \neq \emptyset$.

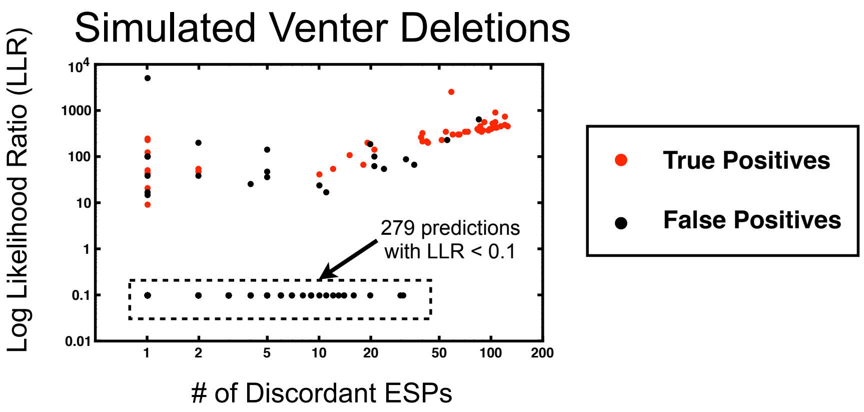

Figure A7: **Likelihood Compared to Number of Supporting Fragments.** We illustrate the number of supporting end-sequenced pairs (ESPs) and likelihood for all GASVPro-HQ predictions with positive likelihood for simulation deletions. As indicated in the Results, the likelihood of a variant as computed by our probabilistic model is a better predictor of the validity of a deletion than the number supporting (ESPs). Importantly, our probabilistic model can accurately predict true variants even with a single discordant ESP.

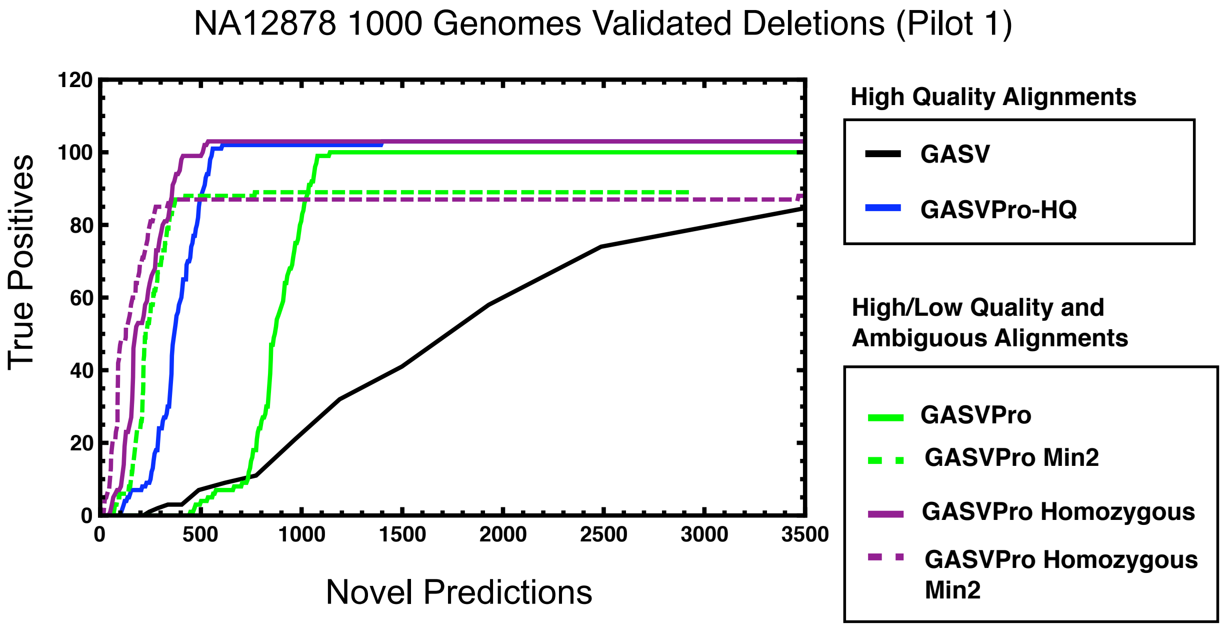

Figure A8: **Homozygous Only Model for Variants on NA12878 Pilot 1 Data.** These ROC curves illustrate a substantial improvement in the sensitivity/specificity of deletion predictions from GASVPro when only the homozygous model for variants was considered. In addition, the number of predictions required to predict the top true positive inversion decreased from 28 to 14 under the homozygous only model for variants. These improvements may indicated a bias in the set of known variants for homozygous events.

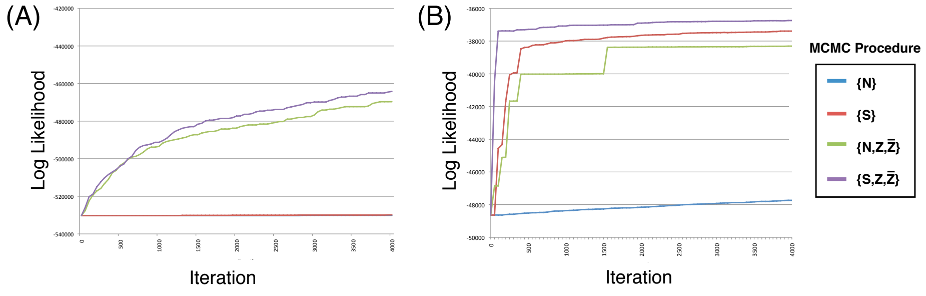

Figure A9: **MCMC Convergence.** We show the log-likelihood of the MCMC sampling procedure for two simulated data sets. In (A) η is chosen to be large, and thus the main factor influencing the $P(M|A)$ is the number of variants with positive support. In these cases convergence is facilitated by moves Z and $\bar{Z}$ which are guaranteed to change the total number of variants. In (B) $P(M|A)$ will depend on both the exponential term as well as the Poisson coverage of each and thus sampling the space is less sensitive to the types of moves chosen.

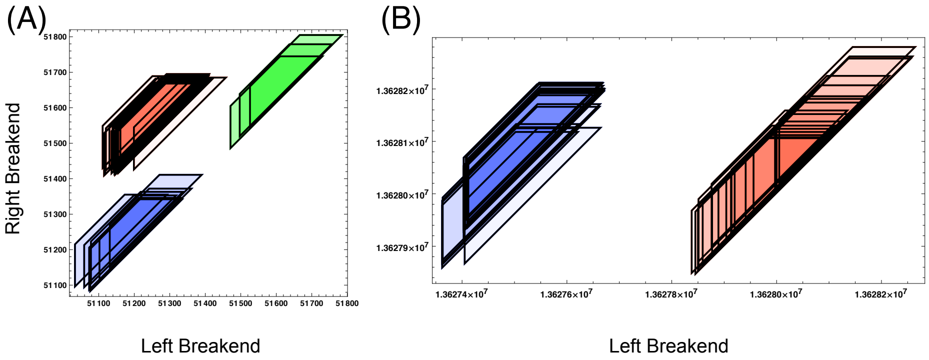

Figure A10: **Differences in Clustering.** Some SV algorithms cluster discordant fragments aggressively by combining fragments that overlap the same genomic interval, whether or not they can support a common novel adjacency. We illustrate this behavior by considering two predicted deletions from Breakdancer on the simulated paired-end data used in this study. Each illustration shows the breakend polygons produced by a single Breakdancer prediction corresponding to (A) 3 GASV clusters (red, blue and green) and (B) 2 GASV clusters (red and blue).

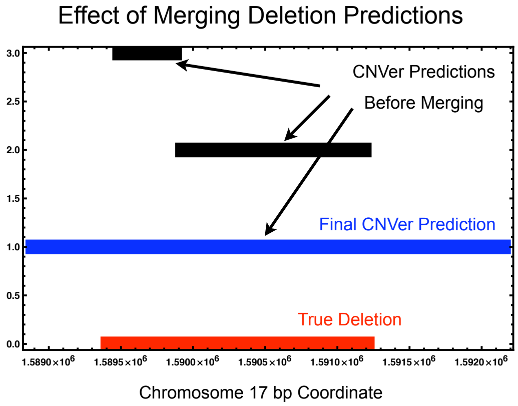

Figure A11: **Effect of Merging Deletion Predictions.** Some SV algorithms merge potential SVs before reporting a final set of predictions. Although such merging may result in a prediction containing a true SV, one effect of merging is a loss of information on the true breakends. We illustrate this behavior by demonstrating the effect of merging deletion predictions in the final steps of CNVer. Although their reported prediction contains the true deletion, the region does not overlap the true deletion in the "double uncertainty" metric.
